# Modelling the spatial and temporal constrains of the GABAergic influence on neuronal excitability

**DOI:** 10.1371/journal.pcbi.1009199

**Published:** 2021-11-12

**Authors:** Aniello Lombardi, Heiko J. Luhmann, Werner Kilb

**Affiliations:** Institute of Physiology, University Medical Center of the Johannes Gutenberg University, Mainz, Germany; University of Pittsburgh, UNITED STATES

## Abstract

GABA (γ-amino butyric acid) is an inhibitory neurotransmitter in the adult brain that can mediate depolarizing responses during development or after neuropathological insults. Under which conditions GABAergic membrane depolarizations are sufficient to impose excitatory effects is hard to predict, as shunting inhibition and GABAergic effects on spatiotemporal filtering of excitatory inputs must be considered. To evaluate at which reversal potential a net excitatory effect was imposed by GABA (E_GABA_^Thr^), we performed a detailed in-silico study using simple neuronal topologies and distinct spatiotemporal relations between GABAergic and glutamatergic inputs.

These simulations revealed for GABAergic synapses located at the soma an E_GABA_^Thr^ close to action potential threshold (E_AP_^Thr^), while with increasing dendritic distance E_GABA_^Thr^ shifted to positive values. The impact of GABA on AMPA-mediated inputs revealed a complex temporal and spatial dependency. E_GABA_^Thr^ depends on the temporal relation between GABA and AMPA inputs, with a striking negative shift in E_GABA_^Thr^ for AMPA inputs appearing after the GABA input. The spatial dependency between GABA and AMPA inputs revealed a complex profile, with E_GABA_^Thr^ being shifted to values negative to E_AP_^Thr^ for AMPA synapses located proximally to the GABA input, while for distally located AMPA synapses the dendritic distance had only a minor effect on E_GABA_^Thr^. For tonic GABAergic conductances E_GABA_^Thr^ was negative to E_AP_^Thr^ over a wide range of g_GABA_^tonic^ values. In summary, these results demonstrate that for several physiologically relevant situations E_GABA_^Thr^ is negative to E_AP_^Thr^, suggesting that depolarizing GABAergic responses can mediate excitatory effects even if E_GABA_ did not reach E_AP_^Thr^.

## Author summary

The neurotransmitter GABA mediates an inhibitory action in the mature brain, while it was found that GABA provokes depolarizations in the immature brain or after neurological insults. It is, however, not clear to which extend these GABAergic depolarizations can contribute to an excitatory effect. In the present manuscript we approached this question with a computational model of a simplified neurons to determine what amount of a GABAergic depolarizing effect, which we quantified by the so called GABA reversal potential (E_GABA_), was required to turn GABAergic inhibition to excitation. The results of our simulations revealed that if GABA was applied alone a GABAergic excitation was induced when E_GABA_ was around the action potential threshold. When GABA was applied together with additional excitatory inputs, which is the physiological situation in the brain, only for spatially and temporally correlated inputs E_GABA_ was close to the action potential threshold. For situations in which the additional excitatory inputs appear after the GABA input or are distant to the GABA input, an excitatory effect of GABA could be observed already at E_GABA_ substantially negative to the action potential threshold. This results indicate that even slightly depolarizing GABA responses, which may be induced during or after neurological insults, can potentially turn GABAergic inhibition into GABAergic excitation.

## 1. Introduction

The neurotransmitter γ-amino butyric acid (GABA) is the major inhibitory neurotransmitter in the adult mammalian brain [1]. GABA regulates the excitation of neurons and is thus essential for e.g. the control of sensory integration, regulation of motor functions, generation of oscillatory activity, and neuronal plasticity [2–4]. GABA mediates its effects via metabotropic GABA_B_ receptors [5] and ionotropic GABA_A_ receptors, ligand-gated anion-channels with a high Cl^−^ permeability and a partial HCO_3_^−^ permeability [6]. The membrane responses caused by GABA_A_ receptor activation thus depend on the reversal potential of GABA receptors (E_GABA_), which is determined mainly by the intracellular Cl^−^ concentration ([Cl^−^]_i_) and to a lesser extent by the HCO_3_^−^ gradient across the membrane [6].

About 30 years ago seminal studies demonstrated that GABA_A_ receptors can mediate depolarizing and excitatory actions in the immature central nervous system [7–9]. This depolarizing GABAergic action reflects differences in [Cl^−^]_i_ homeostasis between immature and adult neurons [10–15]. In particular, low functional expression of a K^+^-Cl^−^ cotransporter (KCC2), which mediates the effective extrusion of Cl^−^ and thus establishes the low [Cl^−^]_i_ required for hyperpolarizing GABAergic membrane responses [16], prevents hyperpolarizing GABA responses in the immature brain. In addition, the inwardly directed Cl^−^ transporter NKCC1 mediates the accumulation of Cl^−^ above passive distribution that underlies the depolarizing membrane responses upon activation of GABA_A_ receptors [17–21]. These depolarizing GABAergic membrane responses play a role in several developmental processes [11,22,23], like neuronal proliferation [24], apoptosis [25], neuronal migration [26], dendro- and synaptogenesis [27], timing of critical periods [28] and the establishment of neuronal circuitry [29]. In addition to early development and of clinical importance, an elevated [Cl^−^]_i_ is also a typical consequence of several neurological disorders in the adult brain, like trauma, stroke or epilepsy and is considered to augment the consequences of such insults [11,30,31].

However, it is important to consider that depolarizing GABA responses do not per se lead to excitatory effects. In fact, the membrane shunting that unescapably accompanies the activation of GABA_A_ receptors can dominate over the excitatory effects of the membrane depolarization [11,32–34]. Theoretical considerations [35,36] suggested that the relation between E_GABA_ and the action potential threshold (E_AP_^Thr^) determine whether activation of GABA_A_ receptors mediates excitatory (E_GABA_ positive to E_Thr_^AP^) or inhibitory (E_GABA_ negative to E_Thr_^AP^) actions. If E_GABA_ was in the voltage range between resting membrane potential and E_Thr_^AP^ the activation of GABA_A_ receptor will induce a depolarizing current, but an excitatory postsynaptic potential (EPSP) that appears during this phase will be dampened in a way that the peak of the EPSP will reach less depolarized values. In case E_GABA_ was positive to E_Thr_^AP^ the GABAergic depolarizing shift dominates over the dampening of the EPSP amplitude, leading to a more depolarized potential at the peak of the EPSP and thus an excitatory effect [36,37]. However, this concept is probably an oversimplification, as within a complex dendritic compartment the local activation of GABAergic conductance influences not only the amplitude of local EPSPs, but also the membrane length and time constants and thus temporal and spatial summation of excitatory synaptic inputs [38,39]. Moreover, the depolarizing effect of GABAergic stimulation outlasts the conductance increase associated with GABA_A_ receptor activation, resulting in a bimodal GABA effect. Close to the initiation of GABAergic responses the shunting effect of the enhanced GABAergic conductance dominate and mediate inhibition. This phase is followed by an excitatory phase dominated by the GABAergic depolarization [40,41]. In addition, E_Thr_^AP^ is a dynamic variable, that depends on the background conductance and the density and adaptation state of voltage-gated Na^+^ channels [10,42,43]. Experimental studies on the effects of GABAergic inputs on neuronal excitability demonstrated for immature neocortical neurons that E_GABA_ required for excitatory GABAergic responses (E_GABA_^Thr^) was close to E_AP_^Thr^ [37], while in immature hippocampal neurons E_GABA_^Thr^ was considerably negative to E_AP_^Thr^ [44]. The observations that (i) the GABA effect can switch from inhibition to excitation for delayed glutamatergic inputs [40], that (ii) GABA inputs in distal dendrites can facilitate neuronal excitability [41], and that (iii) extrasynaptic GABAergic activation mediates an excitatory effect whereas synaptic inputs mediate inhibition [45], also suggest that the reversal potential required for excitation is not only defined by E_AP_^Thr^. This complexity is further supported by recent in-vivo investigations that identified excitatory as well as inhibitory effects in the immature brain [46–48]. In summary, to our knowledge no clear concept is currently available that can explain how E_GABA_ influences GABAergic excitation/inhibition and the effect of GABA on spatiotemporal summation of EPSPs in the dendritic compartment.

Therefore, the present computational study investigates the dependency between E_GABA_ and excitatory and inhibitory consequences of GABA_A_ receptor activation and attempts to establish a general view of the impact of depolarizing GABAergic effects on the excitability of neurons. Our results demonstrate that only for GABAergic synapses located at or close to the soma the difference between E_GABA_ and E_AP_^Thr^ predicts whether GABA has an excitatory or an inhibitory action. The E_GABA_ at which depolarizing GABA actions switch from inhibition to excitation is in most cases negative to E_AP_^Thr^ and depends on the temporal and spatial relation between GABA and AMPA inputs, with a more excitatory effect on AMPA inputs that are delayed or located proximal to GABA inputs. We conclude from our results that GABA can mediate excitatory effects even if E_GABA_ is considerably hyperpolarized to E_AP_^Thr^.

## 2. Results

### 2.1. Simulation of active and passive properties of immature CA3 pyramidal neurons

The parameters used for the models in this study are based on the cellular properties obtained in whole-cell patch-clamp recordings from visually identified CA3 neurons in horizontal hippocampal slices from P4-7 mice. Some parameters of these recordings have been used in our previous report [49]. The analysis of the patch-clamp experiments revealed that the immature CA3 pyramidal neurons had an average resting membrane potential (RMP) of −50.5 ± 1.3 mV, an average input resistance (R_Inp_) of 1.03 ± 0.11 GOhm, and an average membrane capacity (C_M_) of 132.3 ± 33.6 nF (all n = 42). As the passive membrane properties directly influence synaptic integration as well as active properties, like E_AP_^Thr^ or the shape of the action potential (AP), we first adapted the spatial properties and the passive conductance g_pas_ of the ball-and-stick model to emulate the recorded properties. To obtain sufficient similarity for these parameters between the model and the real cells we equipped a ball-and-stick model (soma diameter (d) = 46.6 μm, dendrite length = 1 mm, dendrite diameter = 1 μm) with a passive conductance density (g_pas_) of 1.28*10^−5^ nS/cm^2^ at a reversal potential (E_pas_) of −50.5 mV. This model had a RMP of -50.5 mV, a R_Inp_ of 1.045 GOhm and a C_M_ of 144.4 nF. In some experiments we reduced the topology to a simple ball model (*one node*, *d =* 46.6 μm), without adapting g_pas_, to evaluate the impact of GABA under quasi one-dimensional conditions.

**Fig 1:**
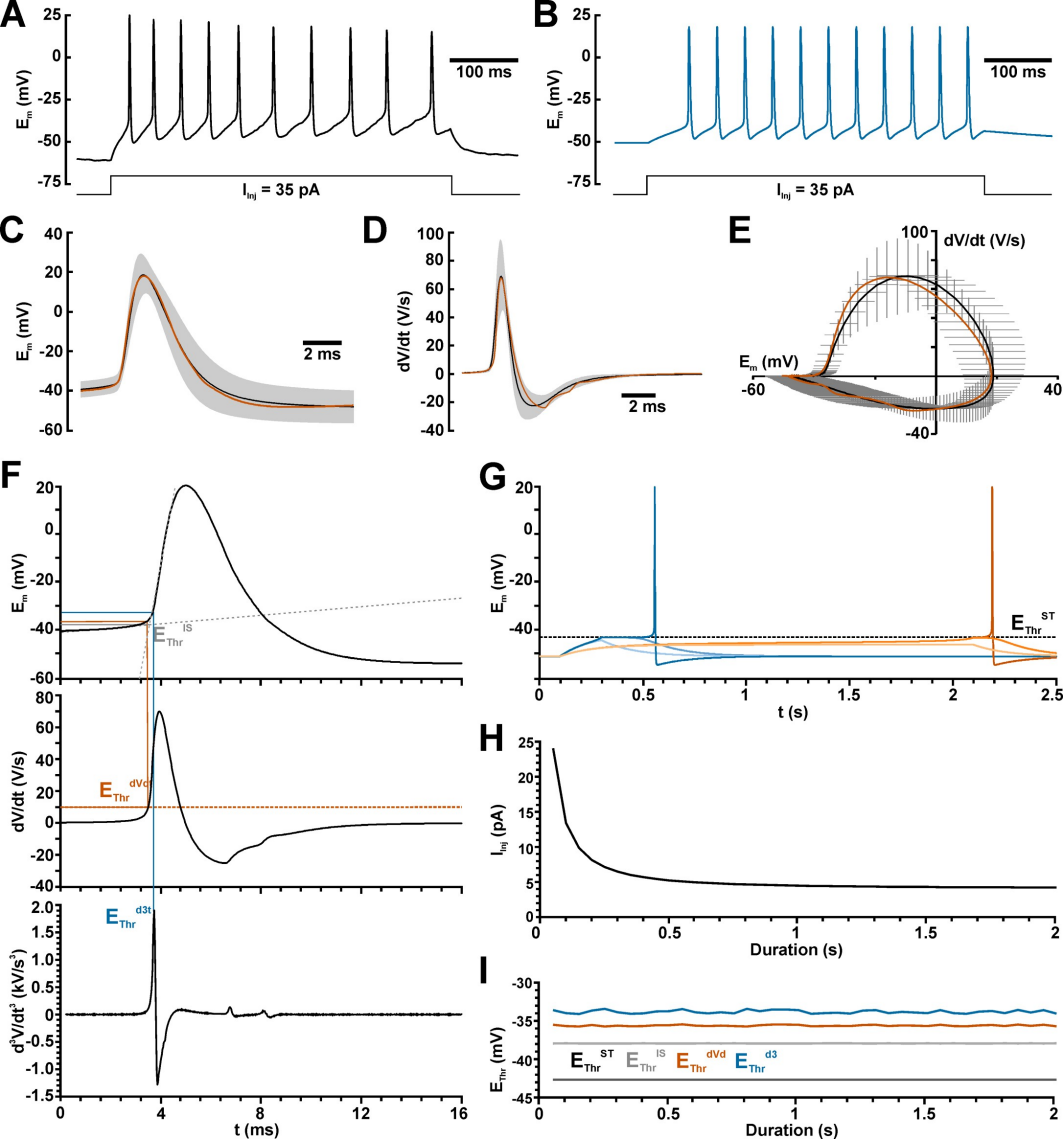
Properties of recorded and simulated action potentials (APs). A: Typical AP train recorded in a CA3 pyramidal neuron upon a current injection of +35 pA. B: AP train simulated in a ball-and-stick model using the modified Neundorf model. C: Average voltage trace of recorded APs (black line = average; gray area ± SEM) and of the simulated AP (orange trace). D: Discharge rate of recorded (black line, gray area) and simulated AP (orange trace). E: Phase plane plot of recorded (whiskers = mean ± SEM) and simulated AP (orange trace). F: Determination of the AP threshold from the intersection of linear voltage fits (E_Thr_^IS^, gray lines), from the timepoint dV/dt reaches the 10 V/s threshold (E_Thr_^dVdt^, orange lines), and from the timepoint d^3^V/dt^3^ reaches the peak value (E_Thr_^d3^, blue lines). G: Determination of the AP threshold at maximal potential of a subthreshold depolarization (E_Thr_^ST^, black lines). Blue traces indicate a 200 ms depolarizing stimulus and orange traces a 2 s stimulus. Dark tones indicate the smallest suprathreshold stimulus, middle tones the largest subthreshold stimulus and light tones a clearly subthreshold stimulus. H: Injection current (I_Inj_) required to elicit an AP at different stimulus durations. I: Values of the various AP threshold parameters for different stimulation durations. Note that AP threshold is independent from the stimulation duration.

With these configurations we next implemented a mechanism that provided APs with properties comparable to the APs recorded in CA3 pyramidal neuron. In particular, we were interested to simulate the AP properties around AP initiation as precisely as possible, because for the main questions of this manuscript we are interested in the E_AP_^Thr^. Since it was not possible to generate a reasonable sharp AP onset with a standard Hodgkin-Huxley (HH) model, we used a model proposed by Naundorf et al. for an optimized spike onset [50]. Using this model with an adjusted parameter set (S4 Table), we were able to simulate APs with a considerable precision (Fig 1A–1E).

Because the relation between E_AP_^Thr^ and E_GABA_ is one major parameter investigated in this study and since no clear definition of the AP threshold has been given [43], we initially used 4 different methods to determine the action potential threshold (Fig 1F): 1.) The AP threshold value E_Thr_^dVdt^ was defined as the potential at which dV/dt first crosses a velocity of 10 V/s [44,51] (Fig 1F orange lines). 2.) E_Thr_^d3^ was defined as the potential at the time point of the first positive peak in d^3^V/dt^3^ [52] (Fig 1F, blue lines). 3.) E_Thr_^IS^ was determined at the intersection between linear regressions of the baseline before the AP and the rising phase of an AP (Fig 1H) [37] (Fig 1F, gray lines). 4.) E_Thr_^ST^ was defined as the maximal potential reached at the strongest subthreshold stimulation (Fig 1G, dashed line), i.e. the minimal potential that did not lead into the regenerative Hodgkin cycle. While the rheobase, i.e. the minimal suprathreshold injection current, demonstrated as expected a hyperbolic increase at shorter stimulus durations and converged to 4.2497 pA (Fig 1H), the distinct E_AP_^Thr^ parameters are virtually independent of the duration of the stimulus (Fig 1I). In the ball model average E_Thr_^dVdt^ was −35.6 mV, average E_Thr_^d3^ was −33.8 mV, average E_Thr_^IS^ was −37.9 mV, and E_Thr_^ST^ converged to −42.8 mV (Fig 1I). When using the ball-and-stick model the rheobase was slightly larger at 6.55 pA, E_Thr_^dVdt^ was −35.5 mV, E_Thr_^d3^ was −33.8 mV, E_Thr_^IS^ was −37.9 mV, and E_Thr_^ST^ converged to −42.2 mV.

For the following simulations between 55 and 63 sweeps were required for each analyzed single parameter (resulting in a total number of ca. 37000 to ca. 500000 single sweeps for each hypothesis, see Materials and Methods for details), thus a time-effective simulation was compulsory. For this purpose, we next evaluated the maximal dt interval required to obtain stable AP responses. This experiment demonstrated that the time course of AP and E_AP_^Thr^ determination remained stable up to a dt of 0.025 ms (S1 Fig). Thus we decided to use a dt of 0.025 ms in the following simulations.

### 2.2. Determination of the threshold for excitatory GABAergic responses

**Fig 2:**
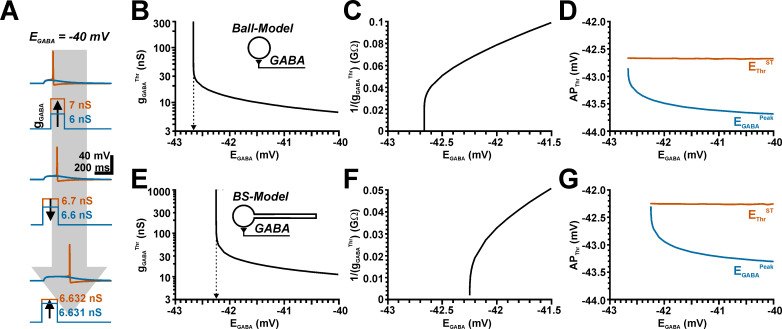
Determination of the threshold conductance at different E_GABA_ enable the identification of E_GABA_ at which responses switch from inhibitory to excitatory (EGABA^Thr^). A: Typical voltage traces illustrating the mechanisms used to determine the threshold g_GABA_ value. For this purpose, g_GABA_ was increased until the first AP was induced (upper panel), then decreased by finer g_GABA_ steps until the AP disappears (middle panel), followed by a subsequent increase in g_GABA_ with finer g_GABA_ steps (lower panel). In total, 6 alternating rounds of increased/decreased g_GABA_ steps were used. The g_GABA_ value required to induce an AP in the last increasing step was considered as threshold (g_GABA_^Thr^). B: Plotting g_GABA_^Thr^ versus E_GABA_ demonstrate that with decreasing E_GABA_ higher g_GABA_^Thr^ values were required, which approximated infinite values. C: A reciprocal plot of g_GABA_^Thr^ enables the precise determination of E_GABA_^Thr^. At E_GABA_ values negative to E_GABA_^Thr^ no action potential could be induced, suggesting a stable GABAergic inhibition. D: The determined AP threshold E_Thr_^ST^ (orange line) is constant over various E_GABA_, whereas the peak potential of the GABAergic depolarization, which was determined at g_GABA_^Thr^ in absence of AP mechanism (E_GABA_^Peak^, blue line) increases with decreasing E_GABA_. Note that the values converged in one point when E_GABA_ reaches E_Thr_^ST^. E-G: Similar plots for a ball-and-stick model. Note that E_GABA_^Thr^ was shifted to less negative values in this configuration.

To identify the reversal potential at which the GABA response switches from inhibitory to excitatory, we first determined the GABAergic conductance that was sufficient to trigger an AP, which was defined as the GABAergic excitation threshold (g_GABA_^Thr^). The value of g_GABA_^Thr^ was determined by systematically increasing the conductance of a simulated GABAergic input until an AP was evoked. To determine this excitation threshold as precisely as possible, we used a multi-step procedure to incrementally confine the threshold conductance (Fig 2A). This procedure was repeated for a whole set of E_GABA_ values (Fig 2B).

In the ball model (*one node*, *d =* 46.6 μm) these systematic simulations demonstrated an obvious hyperbolic increase of g_GABA_^Thr^ when E_GABA_ approaches values below −42.5 mV (Fig 2B). The g_GABA_^Thr^ curve approximated an E_GABA_ of −42.67 mV, which was precisely determined from a reciprocal plot of the g_GABA_^Thr^ values (Fig 2C). Negative to an E_GABA_ of −42.67 mV no action potential could be evoked, regardless of the amount of g_GABA_. These E_GABA_ values thus reflects the threshold, at which GABA actions can mediate a direct excitation and we termed this value “threshold E_GABA_” (E_GABA_^Thr^). Note that this value is in the range of the E_Thr_^ST^ value of −42.8 mV determined in the previous experiments. Since E_AP_^Thr^ is influenced directly by the total membrane conductance, we also determined the amplitude of the GABAergic voltage response under conditions when the AP initiation was blocked (E_GABA_^Peak^) as well as the different E_AP_^Thr^ parameters. These analyses revealed that E_Thr_^d3^ was around −33.5 mV for all E_GABA_. E_Thr_^ST^ was relatively stable around −42.7 mV (Fig 2D). E_GABA_^Peak^ was for higher E_GABA_ around −43.7 mV and showed a positive shift with decreasing E_GABA_ that converged to values of −42.8 mV (Fig 2D).

In summary, these results indicate that GABA acts as excitatory neurotransmitter as long as E_GABA_ is positive to −42.67 mV, which is extremely close to the AP threshold E_Thr_^ST^. This observation is in line with previous predictions that propose exactly this relation between E_AP_^Thr^ and E_GABA_ [35,36]. In addition, our simulations suggest that E_Thr_^ST^ is probably the most relevant definition for E_AP_^Thr^ if the direction of GABA effects should be predicted from the difference between E_GABA_ and E_AP_^Thr^.

Next we performed the same simulation with a ball-and-stick model. These simulations revealed that the g_GABA_^Thr^ curve approximated an E_GABA_ of −42.2 mV (Fig 2E and 2F), which is very close to the E_Thr_^ST^ (−42.2 mV) determined for the ball-and-stick model. E_Thr_^d3^ was around −33.5 mV for all E_GABA_. E_Thr_^ST^ was stable at values around −42.2 mV and converges at low E_GABA_ to −42.25 mV (Fig 2G). E_GABA_^Peak^ was for higher E_GABA_ around −43.4 mV and converged with decreasing E_GABA_ to −42.3 mV (Fig 2G). Thus, E_GABA_^Thr^ for a somatic synapse is still in good agreement with the AP threshold value E_Thr_^ST^ with this slightly more complex neuronal topology.

**Fig 3:**
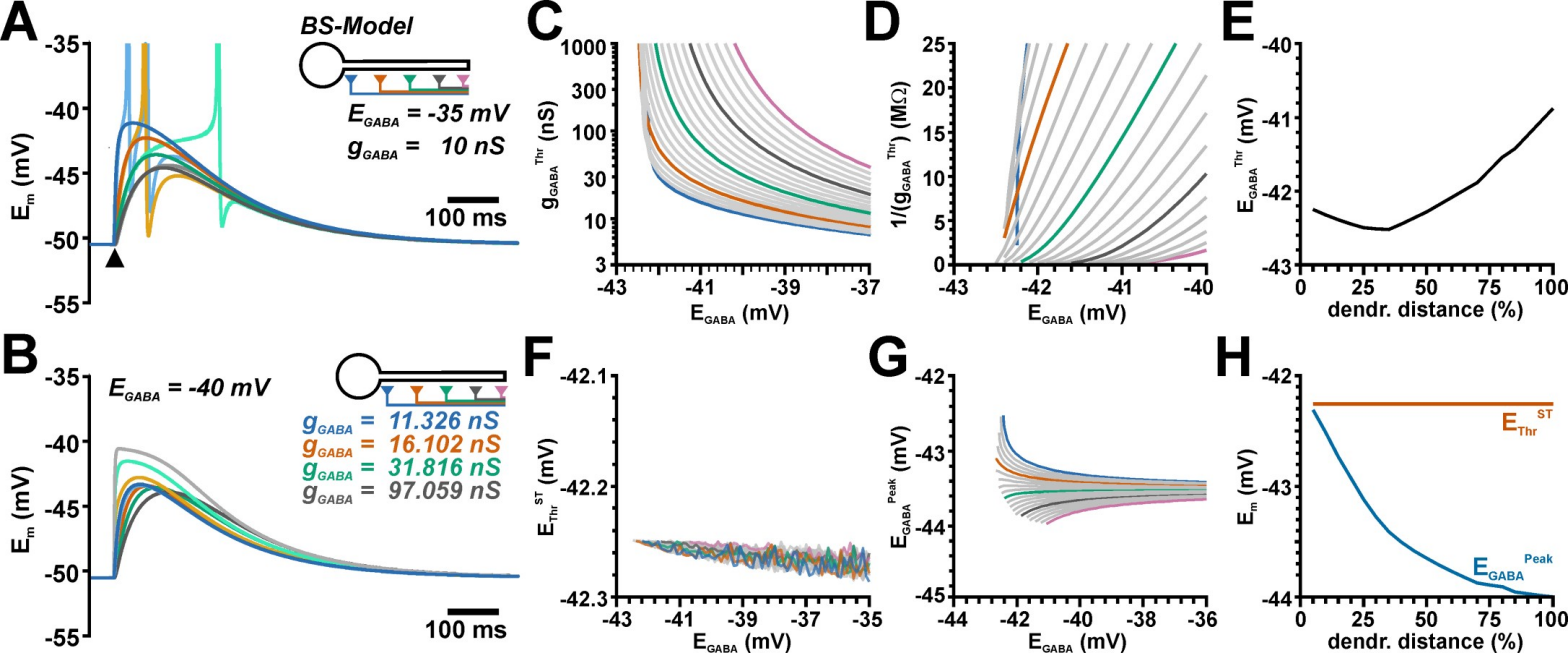
Determination of EGABA^Thr^ at different dendrite positions. A: Simulated voltage traces obtained with the given parameters at different locations as indicated by color code. The light colored traces represent simulation with added AP mechanism. The amplitude of GABA responses clearly depends on the dendritic location. B: Simulated voltage traces for g_GABA_^Thr^ and E_GABA_ of −40 mV at the soma (dark colors) and the synaptic site (light colors). For each location different g_GABA_ (as indicated) had to be used. Note that at distant synapses considerable large g_GABA_ were required, which virtually clamped E_m_ at the synaptic site to E_GABA_. C: Systematic plot of g_GABA_^Thr^ determined at various E_GABA_. The curves were obtained from 20 equidistant positions along the dendrite. The 1^th^, 5^th^, 10^th^, 15^th^ and 20^th^ trace is color-coded as in A for better readability. D: The reciprocal plot of g_GABA_^Thr^ revealed that the curves did not monotonically approach the abscissa. Therefore, E_GABA_^Thr^ was estimated from a linear fit to the last two data-points. E: E_GABA_^Thr^ showed a considerable shift towards depolarized potentials with increasing dendritic distance. F: The AP threshold E_Thr_^ST^ remained rather stable with different E_GABA_ or different synaptic location. G: The peak potential (E_GABA_^Peak^) of the somatic GABAergic depolarization at g_GABA_^Thr^ converges toward E_Thr_^ST^ only for soma-near synapses (dark blue trace). With more distant synapses less depolarized E_GABA_^Peak^ was required. Color code as in C. H: While the average E_Thr_^ST^ (orange line) is stable for all dendritic locations, the E_GABA_^Peak^ at threshold stimulation (blue line) is shifted to more negative values with increasing dendritic distance.

For the next set of experiments, we located a single GABA synapse along the dendrite of the ball-and-stick model and determined E_GABA_^Thr^ for each of these 20 synaptic positions, using the method described above. The considerable conductance and capacitance provided by the dendritic membrane leads, as expected, to a reduced amplitude and a slower time course of the GABAergic PSPs recorded at the dendritic positions (Fig 3A). Accordingly, larger g_GABA_ values were required to trigger APs for more distant dendritic locations of GABAergic inputs (Fig 3B and 3C). At the most distant dendritic positions g_GABA_ values above 100 nS (i.e. more than 100x of g_GABA_ of a single synaptic event [49]) were required to trigger an AP, which virtually clamped the dendritic membrane at the synapse position to E_GABA_ (Fig 3B). A systematic analysis of g_GABA_^Thr^ at different E_GABA_ values illustrated that g_GABA_^Thr^ showed a considerably less steep dependency on E_GABA_ at more distant dendrite positions (Fig 3C). The reciprocal plot of g_GABA_^Thr^ demonstrated that the g_GABA_^Thr^ values did not converge at similar E_GABA_ values for the different synapse locations, but that the curves reached the abscissa at considerable more positive values for distant GABAergic inputs (Fig 3D). Intriguingly, the synapses close to the soma revealed a E_GABA_^Thr^ value close to E_Thr_^ST^, which was shifted to slightly more negative E_GABA_^Thr^ values for dendritic synapses at a distance of ca. 250 μm, and then increased to positive values with additional distance to the soma (Fig 3E). E_GABA_^Peak^, which was determined in the absence of AP mechanisms and reflects the effective voltage fluctuation at the soma and thus the AP initiation site, was shifted to negative potentials at more distant dendritic positions (Fig 3G and 3H), while the position of GABA synapses had no major effect on E_Thr_^ST^ (Fig 3F and 3H). In summary, these simulations revealed that E_GABA_^Th^ is not close to the AP threshold value E_Thr_^ST^ for synapses that are located in the dendrite, but that E_GABA_^Th^ is shifted to more positive values with increasing distance. This observation suggests that for dendritic synapses a more positive E_GABA_ (corresponding to a higher [Cl^−^]_i_) is required to mediate a direct excitatory effect.

### 2.3. Effect of phasic GABAergic inputs on glutamatergic excitation

The previous results demonstrated that only at perisomatic synapses E_GABA_^Thr^ was reached when E_GABA_ was at the action potential threshold E_Thr_^ST^, but that E_GABA_^Thr^ was systematically shifted to positive E_GABA_ at distant synapses in a ball-and-stick model. However, these experiments do not reflect the physiological situation of GABAergic transmission in the brain. First, the threshold conductance g_GABA_^Thr^ determined by these simulations is clearly above physiological values for moderate GABAergic inputs [49,53,54], making a direct excitatory GABAergic input implausible. And second, synaptic activity is characterized by the co-activation of GABA and glutamate receptors [55–57], with the latter constituting the main excitatory drive [58]. Therefore, we next simulated the impact of a GABAergic co-stimulation on glutamatergic synaptic inputs and determined the g_AMPA_ values that were required to trigger an AP. For the present simulation we used a simplified model of glutamatergic synaptic inputs, neglecting NMDA receptors [59,60]. We considered to use the reduced model containing only AMPA and GABA receptors to ease the interpretation of the interactions between both synaptic inputs. The functional relevance of both, GABA_A_ [8,61–63] and AMPA [64–66] receptors from early postnatal stages into adulthood has been clearly demonstrated in the hippocampus and neocortex.

**Fig 4:**
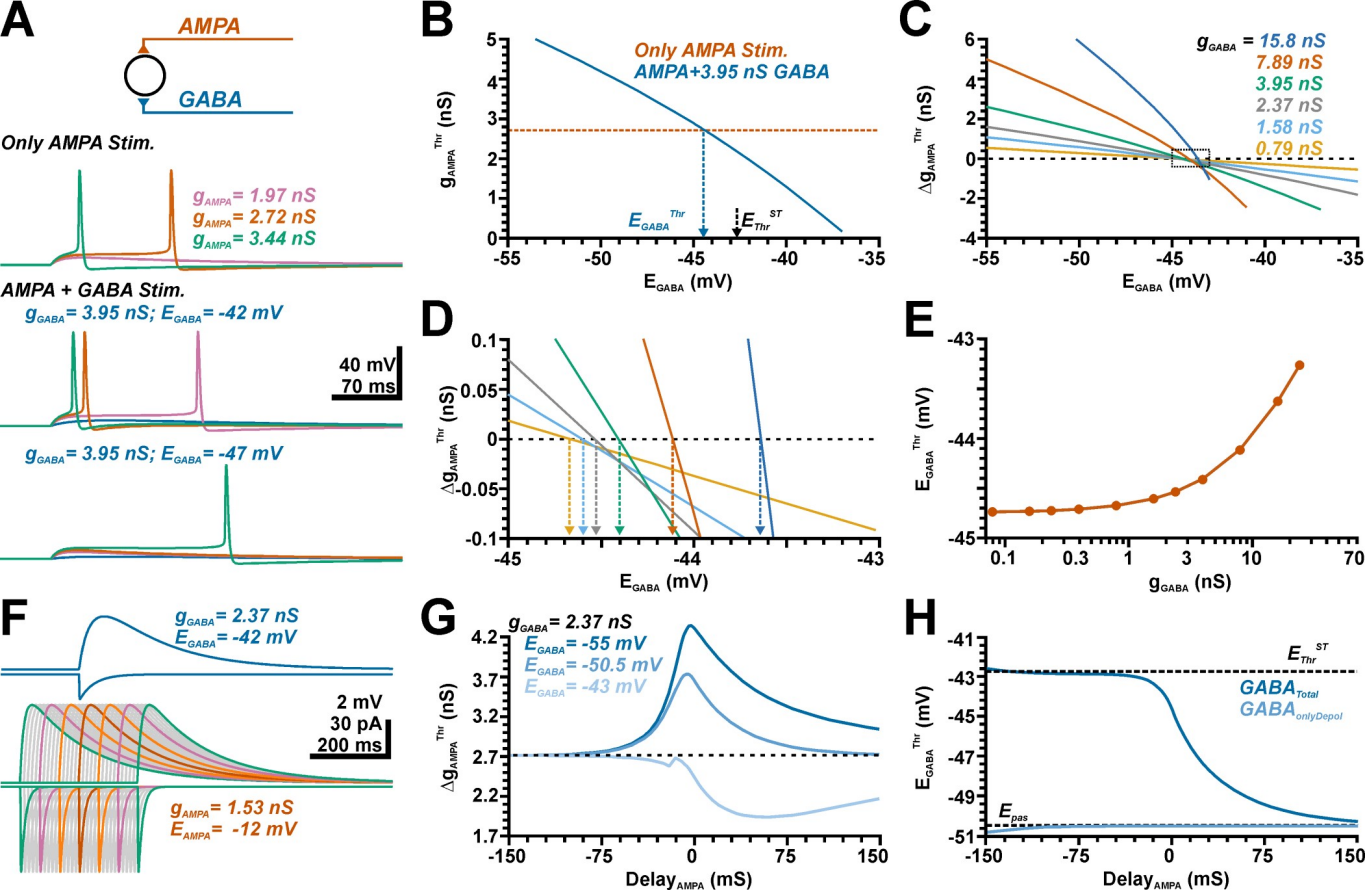
Influence of a GABAergic input at different EGABA^Thr^ on the AMPA receptor-dependent excitation threshold. A: Simulated voltage traces illustrating the membrane responses induced by three different conductances of the AMPA synapse in the absence (top traces) and the presence of a simultaneous GABAergic input at E_GABA_ of −42 mV (middle traces) and −47 mV (lower traces). B: Plot of the minimal g_AMPA_ required to trigger an AP (g_AMPA_^Thr^) versus the E_GABA_ of the synchronous GABA input (g_GABA_ = 3.95 nS). The E_GABA_ value at which this curve intersects with g_AMPA_^Thr^ determined in the absence of GABA (orange line) defines the GABA concentration at which GABA switches from excitatory to inhibitory (E_GABA_^Thr^). C: Plot of Δg_AMPA_^Thr^ versus E_GABA_ for different g_GABA_ values, as indicated in the graph. D: A magnification of the marked area in C allows the determination of E_GABA_^Thr^ for the different g_GABA_, color code as indicated in C. E: Plot of the E_GABA_^Thr^ determined at different g_GABA_. Note that E_GABA_^Thr^ is substantially negative to E_Thr_^ST^ and increases at higher g_GABA_. F: Simulation of membrane currents (downward deflections) and membrane changes (upward deflections) upon a GABAergic (blue traces) and glutamatergic stimulation. The lower traces represent glutamatergic inputs shifted by ± 150 ms in 10 ms steps, each 5th trace was colored for better readability. Note that the depolarization shift outlasts the conductance shift for both inputs. G: Influence of the timing between AMPA and GABA input on Δg_AMPA_^Thr^ determined at 3 exemplary E_GABA_. Note that the maximal inhibitory effect at hyperpolarizing (E_GABA_ = −55 mV) or pure shunting GABAergic inputs (E_GABA_ = −50.5 mV) were observed for synchronous AMPA inputs, while the excitatory influence of GABA at depolarized E_GABA_ of −43 mV was maximal for substantially delayed AMPA inputs. H: Quantification of E_GABA_^Thr^ (dark blue) for different delays between GABA and AMPA inputs. Note that for AMPA inputs preceding GABA inputs E_GABA_^Thr^ was close to the AP threshold, while for AMPA inputs lagging GABA inputs E_GABA_^Thr^ approximated −50.5 mV. The light blue traces represent E_GABA_^Thr^ determined for pure simulated GABAergic depolarizations which persistently results in a E_GABA_^Thr^ close to −50.5 mV.

As in the previous experiments, we varied E_GABA_ to determine E_GABA_^Thr^, which is defined as the E_GABA_ value at which the GABAergic effect shifts from inhibitory (i.e. GABA co-activation requires larger g_AMPA_ to trigger APs) to excitatory action (i.e. GABA co-activation requires less g_AMPA_) (Fig 4A). This effect was quantified as the GABAergic excitability shift (Δg_AMPA_^Thr^), with g_AMPA_^Thr^ describing the g_AMPA_ value sufficient to trigger an AP, and Δg_AMPA_^Thr^ defined as difference in g_AMPA_^Thr^ between conditions with and without GABAergic co-stimulation: [Δg_AMPA_^Thr^ = (g_AMPA_^Th^)_withGABA_—(g_AMPA_^Th^)_w/oGABA_].

In the first set of experiments we simulated the effect of GABA pulses provided synchronously with AMPA inputs in a ball model (Fig 4A) using a constant g_GABA_ of 3.95 nS. These experiments demonstrated that the co-stimulation of a GABAergic input can attenuate or enhance AP triggering upon glutamatergic stimulation, depending on E_GABA_ (Fig 4A). As expected, such a GABA co-stimulation enhanced g_AMPA_^Thr^ at hyperpolarized E_GABA_, while smaller g_AMPA_^Thr^ values were required at more depolarized E_GABA_ (Fig 4B). From the intersection of this g_AMPA_^Thr^ with the g_AMPA_^Thr^ recorded in the absence of GABAergic inputs we determined that E_GABA_^Thr^ amounted to −44.4 mV under this condition (Fig 4B), which is considerable more negative than E_Thr_^ST^ of −42.8 mV determined in the ball model. Additional experiments with different g_GABA_ values revealed that E_GABA_^Thr^ depends on g_GABA_ (Fig 4C– 4E). However, only at rather large g_GABA_ values E_GABA_^Thr^ approached toward values > −44 mV. At lower, physiologically more relevant g_GABA_ values E_GABA_^Thr^ converges to a value of −44.7 mV (Fig 4E). This observation indicates that E_GABA_^Thr^ was consistently lower than E_Thr_^ST^, implying that GABAergic inputs are under these conditions more excitatory than expected from the difference between E_GABA_ and E_AP_^Thr^.

Is has already been proposed that the GABAergic depolarization outlasts the GABAergic currents and can add an additional excitatory drive to neurons [40]. Our simulations replicated this typical behavior, both GABAergic and glutamatergic membrane depolarization outlasted the time course of the respective currents (Fig 4F), To investigate whether the systematic shift of E_GABA_^Thr^ towards more hyperpolarized potentials was indeed caused by the differential impact of GABAergic conductance and GABAergic membrane depolarization on the AMPA-mediated excitation, we systematically advanced or delayed the time point of AMPA inputs (Fig 4F). These simulations revealed that, as expected, the strongest inhibitory effect of a GABAergic input for both hyperpolarizing (at E_GABA_ < RMP) and shunting inhibition (at E_GABA_ = RMP) was observed when it was synchronous to the glutamatergic input (Fig 4G). In contrast, at more depolarized E_GABA_ the maximal excitatory effect occurred when the AMPA input was given about 60 ms after the GABA input (Fig 4G, light trace), i.e. at a time point when the GABAergic conductance virtually ceased but a considerable GABAergic depolarization persisted (Fig 4F, blue traces). A systematic determination of E_GABA_^Thr^ for different delays demonstrated that E_GABA_^Thr^ was relatively stable around −43 mV for APMA inputs that preceded GABA inputs, and was thus close to E_Thr_^ST^ (Fig 4H). In contrast, with increasing delays of the glutamatergic inputs E_GABA_^Thr^ converged to −50.5 mV, i.e. to the RMP determined by the reversal potential of the passive membrane conductance (Fig 4H). In summary, these findings suggest (i) that at preceding AMPA inputs the influence of GABA on this glutamatergic input was dominated by the GABAergic conductance change and thus converged to E_Thr_^ST^ and (ii) that at delayed glutamatergic inputs the influence of GABA on this glutamatergic input was dominated by the GABAergic depolarization.

In the absence of a GABAergic conductance shift each depolarization above −50.5 mV should reduce the distance to the E_AP_^Thr^ and should thus impose an excitatory effect. To verify this hypothesis, we recorded the GABAergic currents at different E_GABA_ and replayed these currents to the modelled neurons via I-clamp, thereby isolating the effect of the GABAergic depolarization from the GABAergic conductance shift. Indeed, these simulations demonstrated that the effect of the pure GABAergic depolarization reversed at an E_GABA_ of −50.5 mV (Fig 4H, light trace).

In summary these experiments demonstrated that the effect of a GABAergic stimulus on glutamatergic synaptic inputs cannot simply be predicted from the difference between E_GABA_ and the E_AP_^Thr^ threshold, but that, depending on the temporal relation between GABAergic and glutamatergic inputs, E_GABA_ is substantially lower than E_AP_^Thr^ and thus GABA acts more excitatory than expected from the E_GABA_ to E_AP_^Thr^ relation.

**Fig 5:**
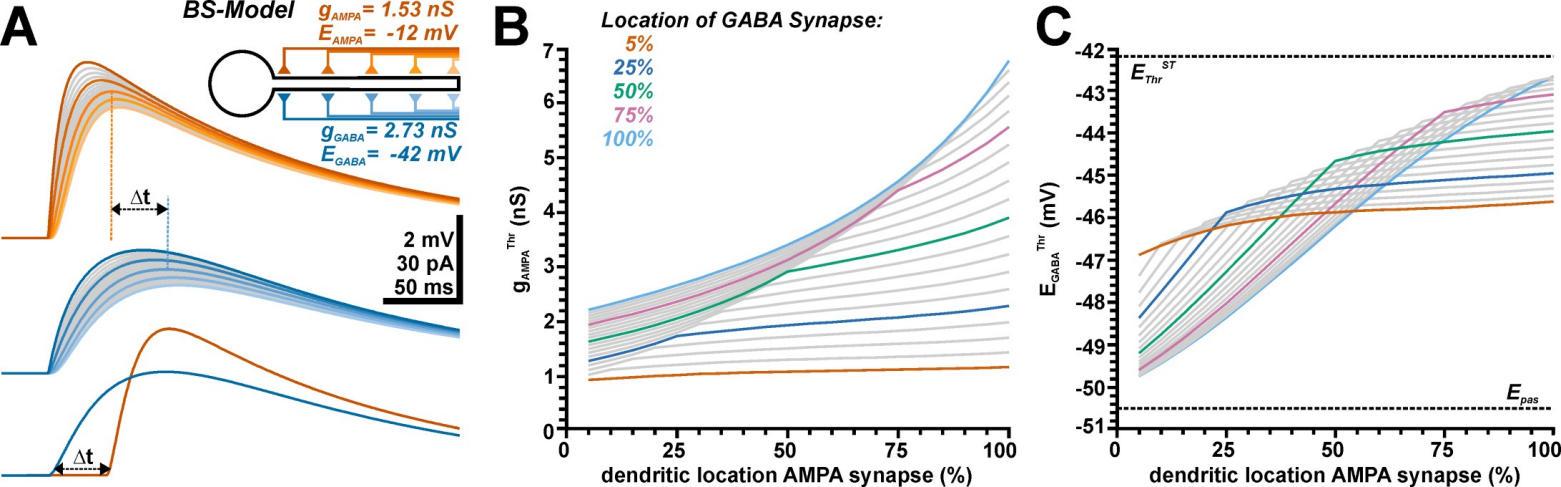
Influence of the spatial relation between the AMPA receptor-dependent and the GABA receptor-dependent synaptic input on gAMPA^Thr^ and EGABA^Thr^. A: Simulated voltage traces illustrating the membrane responses induced by AMPA synapses (orange traces) and by GABA synapses (blue traces) located at different dendritic locations. The colored traces represent synapses at 5%, 25%, 50%, 75% and 100% of the dendritic length, as color-coded in the schematic inset. Note the slower onset kinetics and delayed peak for distant dendritic synapses. The lower traces depict how the delay of GABA and AMPA was adjusted to obtain synchronous peak depolarizations. B: Effect of the dendritic location on g_AMPA_^Thr^ simulated for 20 equidistant positions of the GABAergic synapse (g_GABA_ = 7.89 nS; E_GABA_ = −40 mV). Each line represents the results for one GABA synapse position, the color code identifies every 5^th^ position as indicated. Note the shallow dependency of Δg_AMPA_^Thr^ for proximal and the steep dependency for distal GABA synapses. C: Dependency of E_GABA_^Thr^ on the dendritic positions of AMPA synapses, each line represents the results for one GABA synapse position, with shade coding as in B. Note the shallow location dependency with E_GABA_^Thr^ between ca. −46 and −47 mV for the proximal GABA synapses, while for distal GABA synapses a steep E_GABA_^Thr^ profile between ca. -43 mV and -50 mV was observed.

In the next set of experiments, we evaluated how the spatial relation between GABAergic and glutamatergic inputs affects E_GABA_^Thr^ in a ball-and-stick model. For these simulations, we systematically varied both, GABA and AMPA synapse along the dendrite, using 20 equidistant positions each (Fig 5A), and stimulated both synapses synchronously. Simulations of single inputs revealed that the time course of the glutamatergic and GABAergic depolarizations critically depended on the dendritic location (Fig 5A), which reflect spatial filtering [67]. To prevent that this temporal scattering affects the spatial analysis of GABA/AMPA relations, we determined the maximum of the depolarization in control sweeps performed before each run of the definite simulation for each combination of g_AMPA_, AMPA location, E_GABA_, and GABA location in the absence of an AP mechanism. For the definite simulation sweep the temporal relation between glutamatergic and GABAergic input was shifted such that peak depolarization of GABA and AMPA responses coincided (Fig 5A). To get an impression how a depolarizing GABAergic input at different locations influences g_AMPA_^Thr^, we first varied the position of a GABAergic synapse with a g_GABA_ of 7.89 nS and an E_GABA_ of −40 mV along the dendrite and determined g_AMPA_^Thr^ for each of the 20 AMPA synapse along the dendrite (Fig 5B). These simulations showed, as expected, that (i) g_AMPA_^Thr^ increased with increasing dendritic distance, and (ii) that for a soma-near GABAergic synapse the excitatory effect of GABA was stronger than for distal dendritic locations, as indicated by the larger g_AMPA_^Thr^ required for the distal GABA synapses (Fig 5B). However, we could also demonstrate that (iii) the slope of the g_AMPA_^Thr^ became shallower for AMPA inputs distal to the GABA inputs (Fig 5B), indicating a strong non-linear influence of GABAergic inputs. To determine how the spatial relation between glutamatergic and GABAergic inputs affects E_GABA_^Thr^ we subsequently varied E_GABA_ (at g_GABA_ of 7.89 nS) for all combinations of synaptic positions and determined when Δg_AMPA_^Thr^ switches the direction (Fig 5C). These simulations revealed a complex relation between these three parameters. If the GABAergic synapse was located in the proximal dendrite close to the soma E_GABA_^Thr^ was only weakly dependent on the site of the AMPA synapse and amounted to values between ca. −46 mV and −47 mV (Fig 5C, orange trace). If the GABA synapse was located more distally E_GABA_^Thr^ showed a step dependency on the location of the AMPA synapse for all AMPA synapses located proximally to the GABA synapse, while the shallow dependency was maintained for the more distal synapses (Fig 5C). Under this condition E_GABA_^Thr^ approached −50 mV for proximal AMPA synapses, i.e. when both synapses were 950 μm apart and thus the GABAergic depolarization dominated over the more local shunting effect (see light blue trace in Fig 5C). In contrast, for distal AMPA and GABA synapses, which represent spatially correlated inputs distant from the AP initiation zone, E_GABA_^Thr^ approached E_Thr_^ST^ (Fig 5C).

GABAergic synapses are not only located in the somatodendritic compartment, but can also be found in the axon initial segment [68]. Intriguingly at these synapses the developmental profile of Cl^-^ transporter expression was extended until peri-adolescent periods [69], resulting in a substantially depolarized E_GABA_ of this GABA synapse with putative excitatory effect [70,71]. To estimate whether the strategic location of this synapse implies a specific dependency on E_GABA_, we also simulated a simple topology that includes an axon with a GABAergic synapse in its initial segment (S2 Fig). For somatically located AMPA inputs these simulations revealed that E_GABA_^Thr^ amounted to −44.1 mV (S2A and S2B Fig) for a physiological g_GABA_ of 0.789 nS. With increasing g_GABA_ E_GABA_^Thr^ was obtained at more depolarized values (S2B Fig). For dendritic localizations of AMPA synapses we found that E_GABA_^Thr^ was shifted to more negative values with increasing distance of the synapses from the soma (S2C Fig). However, these E_GABA_^Thr^ values were under all conditions less than 1 mV depolarized to simulations with identical parameters but with a somatic localization of the GABA synapse. Thus with regard to their E_GABA_ dependency GABA synapses in the axon initial segment are comparable to somatic GABA synapses.

In summary, these results demonstrate that both, the spatial relation between GABAergic and glutamatergic synapses as well as the location of the GABA synapse influences E_GABA_^Thr^. However, only for spatially correlated inputs at distal dendrites E_GABA_^Thr^ was close to the E_AP_^Thr^. With increasing distance between both synapses and with a closer approximation of the GABA synapse to the soma, E_GABA_^Thr^ was shifted to more negative values, again indicating that GABA mediates a more prominent excitatory action than expected from the difference between E_GABA_ and E_AP_^Thr^.

### 2.4. Effect of tonic GABAergic inputs on glutamatergic excitation

**Fig 6:**
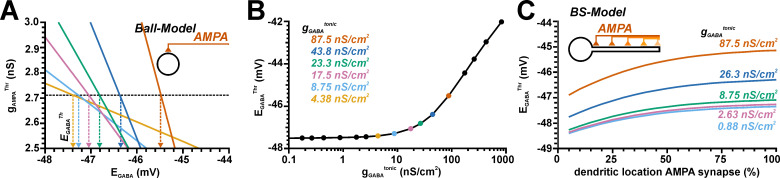
Influence of tonic GABAergic conductances on the AMPA receptor-dependent excitability in a simple ball and a ball-and-stick model. A: Plot of g_GABA_^Thr^ at different E_GABA_. The colored lines represent different tonic g_GABA_ values as indicated in B. The increased slope of the curves with higher g_GABA_^tonic^ illustrates the higher inhibitory effect under this conditions. From the intersection of the plots with the g_AMPA_^Thr^ value obtained in the absence of tonic GABA (dashed line) the E_GABA_^Thr^ values were determined. B: Plot of E_GABA_^Thr^ determined at different g_GABA_^tonic^. Note that E_GABA_^Thr^ is negative to E_Thr_^ST^ for g_GABA_^tonic^ < ca. 300 nS/cm^2^. C: Influence of different dendritic locations of AMPA synapses in a ball-and-stick model on the AMPA receptor-dependent excitability determined for different g_GABA_^tonic^. Note the substantial shift of E_GABA_^Thr^ to positive values with more distant AMPA synapses and the systematic depolarized shift with increasing g_GABA_^tonic^.

GABA influences neuronal excitability not only via synaptic inputs, but also extrasynaptic, tonic GABAergic currents substantially contribute to the GABAergic effects [72,73] and can mediate even excitation during development [45]. Therefore, we next analyzed how a tonic GABAergic conductance (g_GABA_^tonic^) influences g_AMPA_^Thr^ and E_GABA_^Thr^ in a ball model (Fig 6A), using a g_GABA_^tonic^ between 87.5 pS/cm^2^ and 8.75 μS/cm^2^, corresponding to values from 1/100 to 1000 times of the experimentally determined tonic GABA conductance of 8.75 nS/cm^2^ [53]. These experiments demonstrated, that g_GABA_^tonic^ can attenuate or enhance AP induction by AMPA synapses, depending on E_GABA_. As expected, the slope of the GABAergic influence increased with g_GABA_^tonic^ (Fig 6A). And as expected, tonic GABAergic conductance enhanced g_AMPA_^Thr^ at hyperpolarized E_GABA_, while smaller g_AMPA_^Thr^ values were required at more depolarizied E_GABA_ (Fig 6A). From the intersection of these g_AMPA_^Thr^ with the basal g_AMPA_^Thr^ (obtained in the absence of tonic GABA), E_GABA_^Thr^ was determined (Fig 6B). Notably, these E_GABA_^Thr^ were rather constant at ca. −47.5 mV within a wide range of g_GABA_^tonic^, spanning from 0.01 to about the experimentally determined g_GABA_^tonic^ value. Only at very high g_GABA_^tonic^ of > 100 nS/cm^2^ E_GABA_^Thr^ approached E_Thr_^ST^ (which is under this conditions shifted to positive values due to the massively enhanced total membrane conductance). In summary, these results indicate that tonic GABAergic conductances can mediate an excitatory effect even if E_GABA_ was substantially negative to E_AP_^Thr^.

In addition, we investigated how the E_GABA_ of g_GABA_^tonic^ affects the excitation generated by AMPA synapses located along the dendrite in a ball-and-stick model (Fig 6C). These simulations revealed that E_GABA_^Thr^ was systematically shifted to positive values for distal AMPA synapses and that E_GABA_^Thr^ was more positive for larger g_GABA_^tonic^ at all dendritic positions (Fig 6C). These observations suggest that a tonic GABAergic conductance mediates an excitatory effect even at E_GABA_ that is substantially negative to E_AP_^Thr^, but that an inhibitory effect of tonic GABAergic conductance is higher at distal AMPA-mediated inputs.

### 2.5. Effects of GABAergic inputs on glutamatergic excitation in neurons with a realistic dendritic morphology

**Fig 7:**
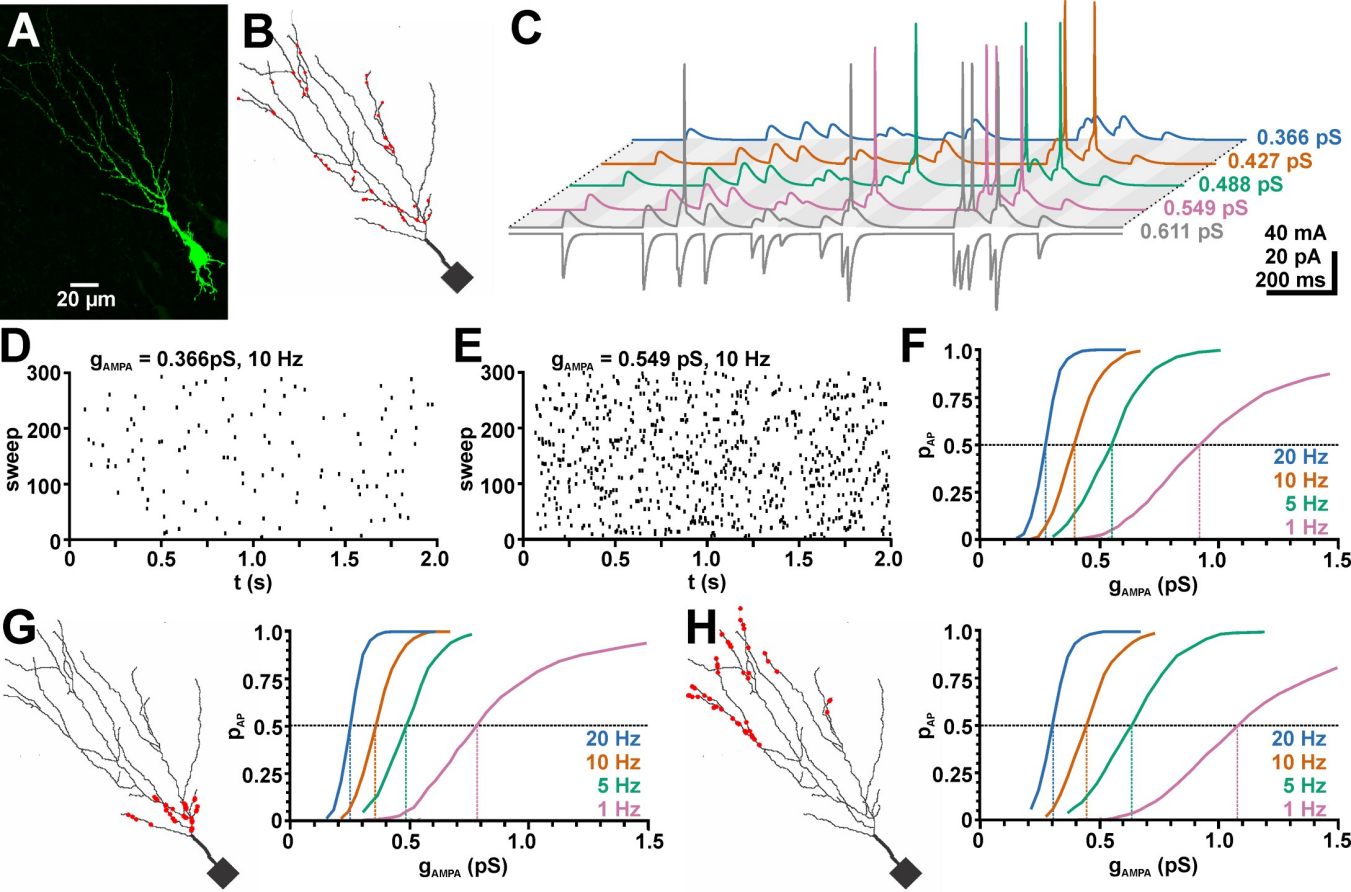
Effect of AMPA mediated synaptic inputs on the excitability of a neuron with a dendritic topology derived from a reconstructed CA3 neuron. A: Biocytin-FITC microfluorescence image of a CA3 pyramidal neuron. B. 2D model of this neuron. The red dots mark the location of randomly distributed AMPA synapses. C: The bottom trace illustrates the synaptic currents at a g_AMPA_ of 0.611 pS and the color coded top traces the respective membrane responses at 5 different g_AMPA_ for a stimulation frequency of 10 Hz. D, E: Raster plots depicting the occurrence of APs in 300 sweeps using random AMPA stimuli at a g_AMPA_ of 0.366 pS and 0.549 pS, respectively. F: Probability for the occurrence of at least one AP (p_AP_) at different g_AMPA_. The dashed vertical lines indicate the g_AMPA_ values at which p_AP_ was 0.5. G: A slight excitatory shift was induced when AMPA synapses were restricted to the proximal dendrite. H: If AMPA synapses were restricted to the distal dendrite the spike probability function was shifted to larger g_AMPA_ values. The g_AMPA_ values obtained in these simulations were used for the determination of the GABAergic effects.

So far our results demonstrated that the impact of E_GABA_ critically depends on the location of GABAergic synapses and their distance to glutamatergic inputs. However, since our previous models represent rather simplified morphological conditions, we next attempted to estimate the impact of distinct E_GABA_ values under more realistic conditions. For this purpose, we used a neuronal topology we derived from a reconstructed, biocytin-labeled CA3 pyramidal neuron (Fig 7A and 7B) [49], that we already utilized in previous in-silico studies [74,75]. Using this topology, we estimated the effect of GABA synapses at different E_GABA_ from the impact of GABAergic input on the spike probability upon glutamatergic inputs. GABA and AMPA synapses were randomly distributed across the dendritic compartment and each synapse was stimulated at a random time point during the 2 s stimulation interval (Fig 7B and 7C). The spike probability (p_AP_) was determined from 999 single sweeps, each with a new distribution of synapse location and stimulus times.

We first determined the *g*_*AMPA*_ values required to mediate a p_AP_ of 50% (g_AMPA_^50^) in the absence of GABAergic synapses (Fig 7). From these simulations, we obtained g_AMPA_^50^ values between 0.92 nS and 0.275 nS for stimulation frequencies between 1 Hz and 20 Hz (Fig 7F, S1 Table). In order to reveal the spatial components of GABAergic inhibition in further experiments, we repeated this g_AMPA_^50^ determination also for AMPA receptors that were distributed only in distal or proximal dendrites (Fig 7G and 7H, S1 Table).

**Fig 8:**
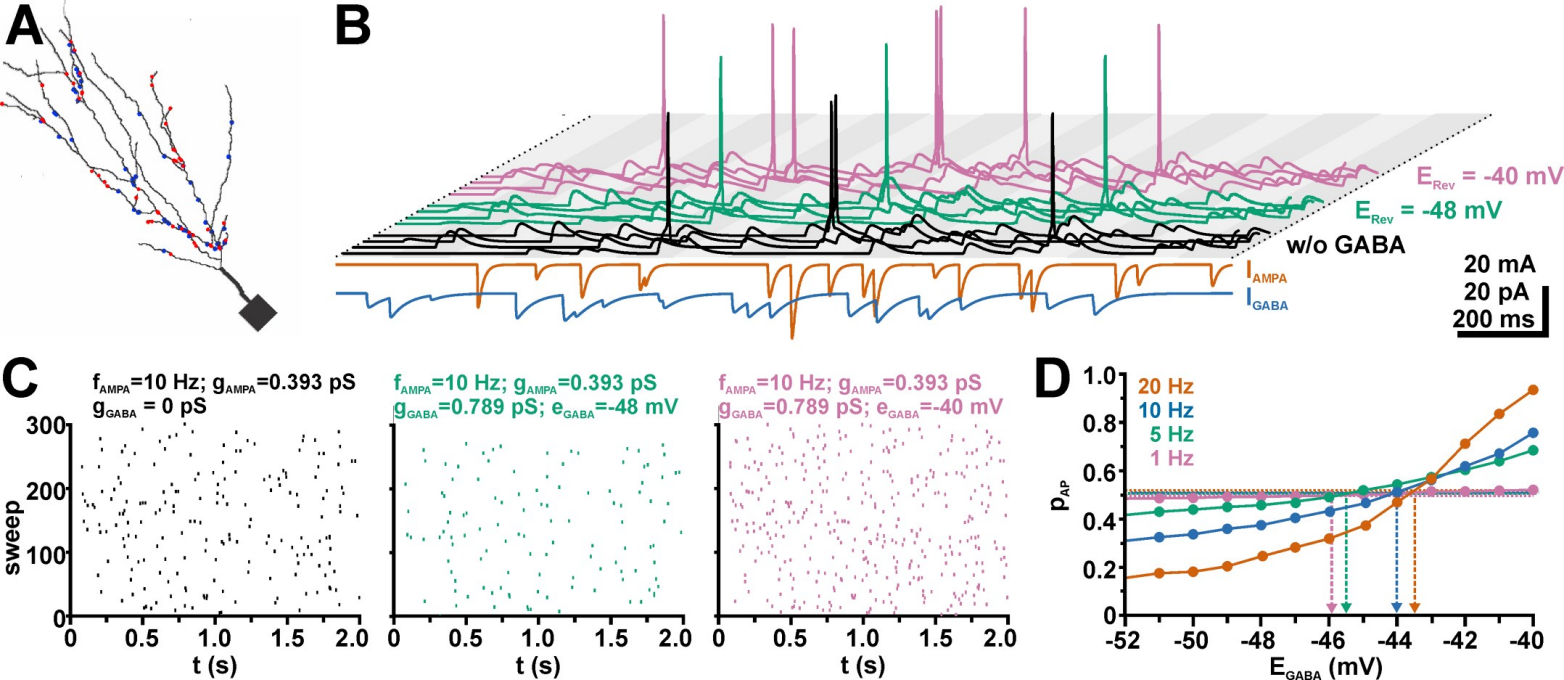
Effect of GABAergic synaptic inputs on the excitability of a neuron with a dendritic topology derived from a reconstructed CA3 neuron. A: 2D model of a CA3 neuron. The red dots mark the location of randomly distributed AMPA synapses and the blue dots that of randomly distributed GABA synapses. B: The bottom traces illustrate the synaptic glutamatergic (0.516 nS, orange) and GABAergic (0.789 nS; blue) currents. The top traces illustrate the voltage responses of 3 sweep obtained for only AMPA receptor stimulation (black) or with a GABAergic co-stimulation at E_GABA_ of either −46 mV (orange) or −40 mV (blue). C: Raster plots depicting the occurrence of APs in 300 sweeps using only AMPA receptor stimulation (left panel) or GABA co-stimulation at E_GABA_ of −46 mV (middle panel) or −40 mV (right panel). Note the inhibitory effect of GABA co-stimulation at E_GABA_ of −46 mV and the excitatory effect at −40 mV. D: AP probability (p_AP_) determined with frequencies of 1 Hz, 5 Hz, 10 Hz, and 20 Hz for a g_GABA_ of 0.789 nS at different E_GABA_. Note the sigmoidal dependency and that p_AP_ reverses at E_GABA_ between −43.5 mV and −45.9 mV.

Next we randomly distributed and stimulated GABA and AMPA synapses across the dendritic compartment (Fig 8A), using the previously determined g_AMPA_^50^ values as gain of the AMPA inputs. As expected, these simulations revealed that random co-stimulation with GABAergic synapses reduced p_AP_ at negative E_GABA_, while p_AP_ was enhanced at more positive E_GABA_ (Fig 8B and 8C). The E_GABA_^Thr^ values obtained from the intersection of the p_AP_ curve with the p_AP_ value of AMPA inputs only (which was close to 0.5, but not exactly at this value, see the dashed lines in Fig 8D), was for a frequency of 20 Hz at −43.5 mV (Fig 8D, S2 Table), and thus only ca 1 mV positive to the E_Thr_^ST^ value of −42.8 mV. At lower frequencies, E_GABA_^Thr^ was substantially more negative and reached −45.9 mV at 1 Hz (Fig 8D, S2 Table), indicating that under these conditions an excitatory effect of GABA can be observed already at lower [Cl^-^]_i_.

**Fig 9:**
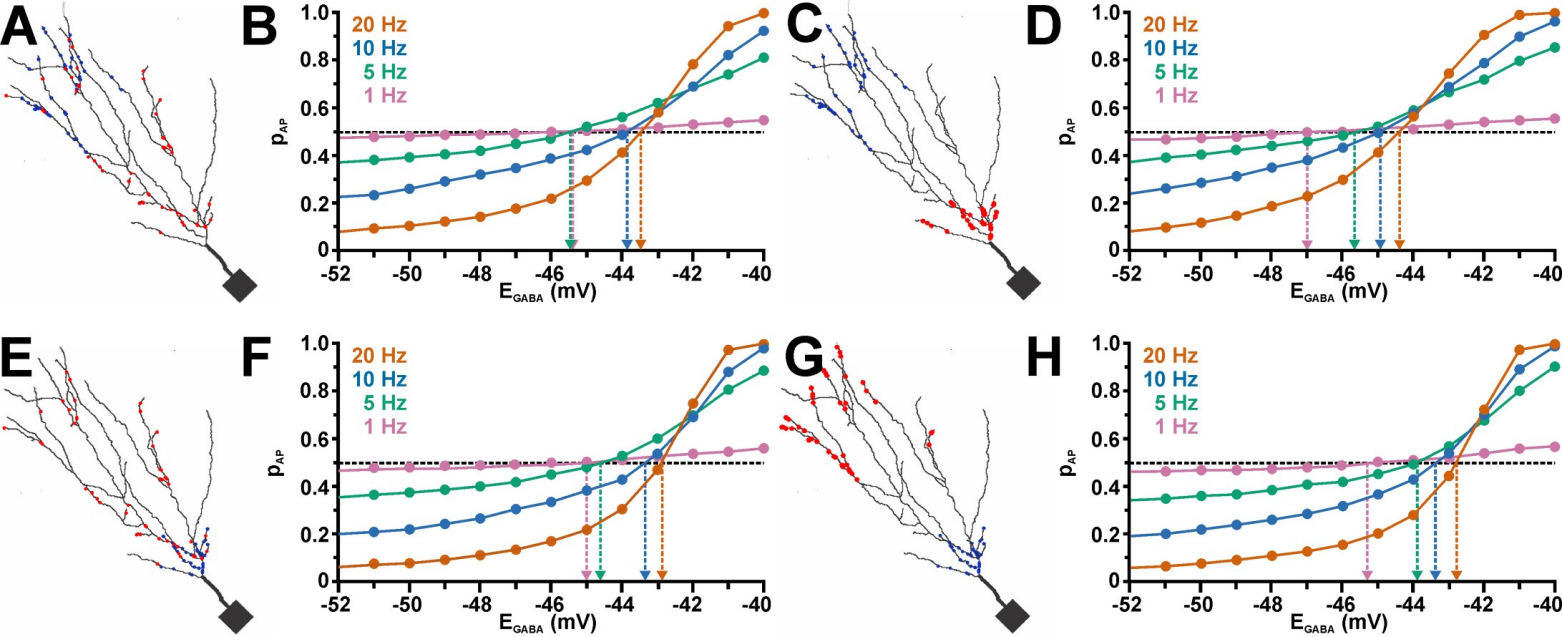
Effect of the site of GABAergic synaptic inputs on EGABA^Thr^. A: 2D model illustrating the random distribution of AMPA synapses (red dots), while the GABA synapses (blue dots) were restricted to distal dendrites. B: p_AP_ vs. E_GABA_ plot for a g_GABA_ of 2.27 pS at 1 Hz, 5 Hz, 10 Hz, and 20 Hz, as identified by the colors. Note that the curve was comparable to the results obtained with a random distribution of GABA synapses (Fig 8D). C, D: As in A and B but for proximal AMPA synapses and distal GABA synapses. Note that E_GABA_^Thr^ was systematically shifted to negative values. E, F: As in A and B but for globally distributed AMPA synapses and proximal GABA synapses. Under this condition a slight positive shift in E_GABA_^Thr^ was observed for higher frequencies. G, H: As in A and B but for distal AMPA synapses and proximal GABA synapses. Note that the E_GABA_^Thr^ values were comparable to F.

Since GABAergic synapses showed a non-homogenous distribution in neurons [68], we also simulated conditions in which the GABA synapses were randomly distributed either in the most distal or the most proximal dendrites (Fig 9). In these simulations we placed the AMPA synapses either throughout the whole dendrite (Fig 9A and 9E), or opposing to the GABA synapse location (Fig 9C and 9G). These simulations revealed that with distally located GABA synapses E_GABA_^Thr^ was slightly shifted to negative values by ca. 0.1–0.5 mV (Fig 9A and 9B; S2 Table). This effect was not systematically altered when the AMPA synapses were restricted to the proximal dendrite (Fig 9C and 9D; S2 Table). Localization of GABA synapses in the proximal dendrites shifted E_GABA_^Thr^ slightly towards more positive values (Fig 9E and 9F; S2 Table). For higher frequencies this effect was even more pronounced when the AMPA synapses were restricted to distal dendrites. E.g. at 20 Hz stimulation with a gGABA of 2.27 nA E_GABA_^Thr^ amounted to −43 mV for evenly distributed AMPA and GABA synapses, which was shifted to −42.8 mV if the GABA synapses were restricted to proximal dendrites, and to −42.7 mV if in addition the AMPA synapses were restricted to the distal dendrites (S2 Table).

In summary, these results demonstrate that for uncorrelated high frequency inputs E_GABA_^Thr^ was close to E_Thr_^ST^, while it was more negative for lower frequencies. A slight but systematical E_GABA_^Thr^ shift in negative direction was observed for distal GABA inputs, while more proximal inputs brought E_GABA_^Thr^ even closer to E_Thr_^ST^. These observations suggest that for high frequency inputs GABA mediates a stable inhibitory effect as long as E_GABA_ is negative to E_AP_^Thr^. For lower frequencies even less depolarized E_GABA_ can mediate an excitatory effect. Although there is a systematic effect of the location of GABA receptors on E_GABA_^Thr^, only a small shift towards more stable inhibitory conditions for proximally located synapses can be deduced from these simulations.

**Fig 10:**
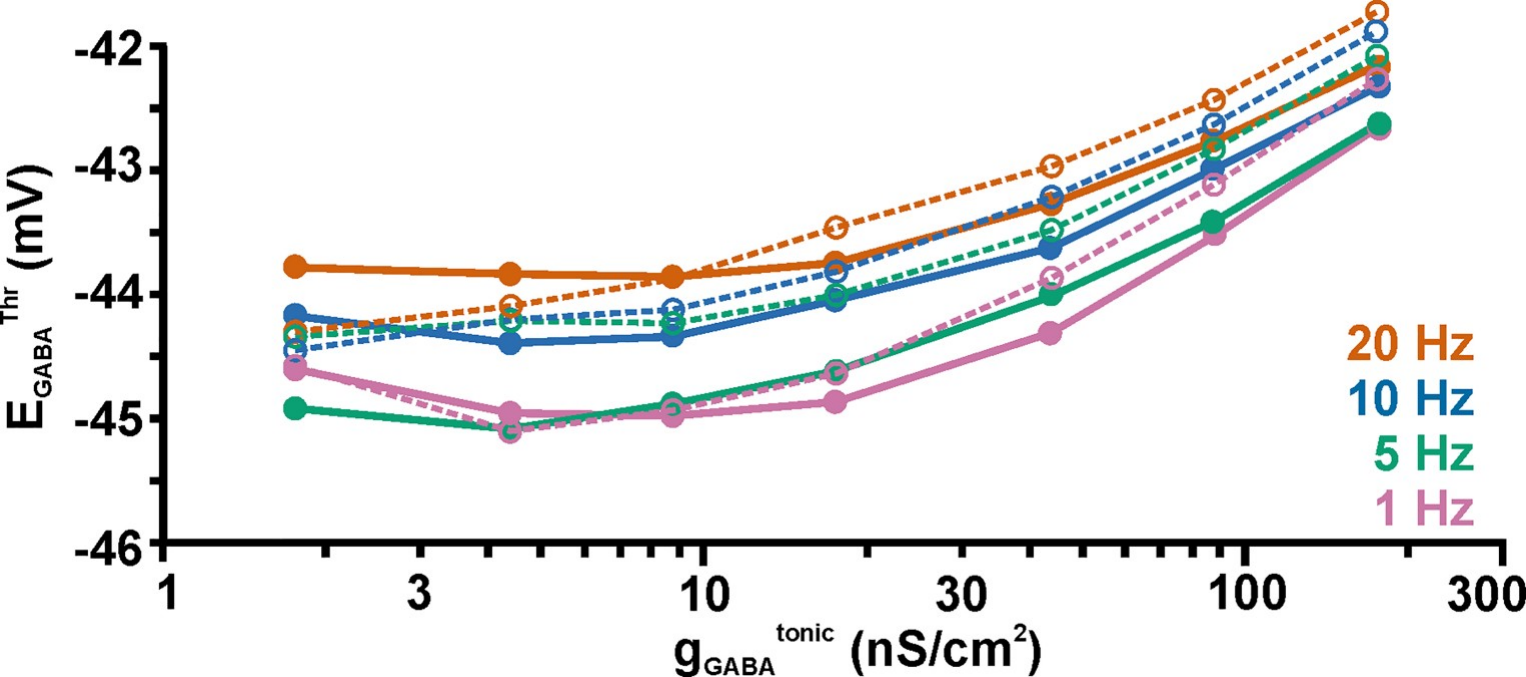
Effect of tonic GABAergic conductances on EGABA^Thr^ in a neuron with a dendritic topology derived from a reconstructed CA3 neuron. The graph displays E_GABA_^Thr^ values obtained for different g_GABA_^tonic^ and f_AMPA_. Dashed lines/open symbols indicate E_GABA_^Thr^ under conditions when AMPA receptors were restricted to the distal dendrites. Note that at physiological g_GABA_^tonic^ values of 8.75 nS/cm^2^, E_GABA_^Thr^ amounted to ca. -45 mV and was shifted to positive values with increasing frequencies. Augmenting g_GABA_^tonic^ systematically shifted E_GABA_^Thr^ towards E_AP_^Thr^. Note that the E_GABA_^Th^ values were slightly shifted to more positive values when the synapses were restricted to the distal compartment, however, this effect was negligible for physiological g_GABA_^tonic^. The intersection between the 1 Hz and 5 Hz curves at low g_GABA_^tonic^ was most probably caused by inaccuracies with the exact determination of changes in the spike probability induced by such small conductance shifts.

Finally, we also simulated the effect of tonic GABAergic currents on E_GABA_^Thr^ with this more realistic dendritic topology. For this purpose, we stimulated randomly distributed AMPA synapses at random time points using different frequencies between 1 Hz and 20 Hz, while simulating evenly distributed tonic GABAergic currents with different conductance densities g_GABA_^tonic^. The effect on E_GABA_^Thr^ was again quantified from the E_GABA_ value at which the shift in the p_AP_ curve upon random AMPA inputs changes from inhibition (< 0.5) to excitation (> 0.5). This simulation revealed that for the physiologically determined g_GABA_^tonic^ value of 8.75 nS/cm^2^ [53], E_GABA_^Thr^ was at −45 mV for 1 Hz stimulation frequency and was shifted in depolarizing direction with increasing frequency of AMPA inputs (Fig 10, S3 Table). At higher g_GABA_^tonic^, E_GABA_^Thr^ was systematic shifted to positive values, approximating E_AP_^Thr^ (Fig 10). For simulations in which the AMPA receptors were restricted to the distal dendrites comparable results were observed (Fig 10). In summary, these results suggest that under consideration of a more realistic dendritic compartment and random AMPA inputs, tonic GABAergic currents reliably mediate an inhibitory effect as long as E_GABA_ is less than ca. 1 mV depolarized to E_AP_^Thr^.

## 3. Discussion

Experimental findings indicate that [Cl^−^]_i_ and [HCO_3_^−^]_i_ are dynamically shifted during early brain development, upon massive GABAergic activity and after pathophysiological insults [10,15,76]. Thus it became evident that GABA can have depolarizing actions [8,13] and this raised the question under which conditions the activation of GABA_A_ receptors can mediate an excitatory effect. Theoretical considerations suggested that GABA_A_ receptor activation permits an inhibitory effect as long as E_GABA_ was below E_Thr_^AP^ [35,36]. However, this consideration just reflects a quasi one-dimensional situation and ignores the temporal and spatial components of GABAergic membrane responses as well as the restriction imposed by the passive membrane properties within more complex neuronal topologies [38–40]. Because the exact role of GABA on the excitation/inhibition threshold is therefore hard to predict from such theoretical assumptions, we performed a detailed in-silico study using primary a simple neuronal topology and distinct spatiotemporal relations between GABAergic and glutamatergic inputs to evaluate at which E_GABA_ values the net GABA effect switches from inhibitory to excitatory. In these simulations we were able to demonstrate that (i) for GABAergic synapses located close to the AP initiation zone (AIP) the difference between E_GABA_ and E_AP_^Thr^ indeed reliably predicts whether GABA has an excitatory or inhibitory action. (ii) The threshold GABA reversal potential (E_GABA_^Thr^) was in this case close to the E_AP_^Thr^ defined by the maximal subthreshold current injection (E_Thr_^ST^). (iii) E_GABA_^Thr^ was systematically shifted to positive values with increasing distance between the GABA synapse and the AIP. (iv) An excitatory effect of GABA inputs on synchronous AMPA mediated inputs was observed when E_GABA_ was above −44.9 mV, and thus consistently hyperpolarized to E_AP_^Thr^. (v) E_GABA_^Thr^ critically depends on the temporal relation between GABA and AMPA inputs, with a striking excitatory effect on AMPA-mediated inputs appearing after the GABA input. (vi) The spatial relation between GABAergic and AMPA-mediated inputs critically influences E_GABA_^Thr^, with E_GABA_^Thr^ systematically being shifted to values negative to E_AP_^Thr^ for AMPA synapses located proximally to the GABA input. (vii) The E_GABA_^Thr^ values for GABA synapses in the axon initial compartment were comparable to somatic GABA synapses. (viii) For tonic GABAergic conductances, E_GABA_^Thr^ was systematically negative to E_AP_^Thr^ over a wide range of g_GABA_^tonic^ values spanning the physiological range. (ix) Simulations using a neuron model with a realistic dendritic compartment revealed that E_AP_^Thr^ was only for high frequency inputs close to E_AP_^Thr^, but was slightly shifted to hyperpolarized values with lower frequencies and a more distal localization of GABA synapses. In summary, these results demonstrate that only for very restricted conditions the GABAergic effects switch from excitation to inhibition when E_GABA_ was at E_AP_^Thr^. Under several physiologically relevant conditions, E_GABA_^Thr^ was negative to E_AP_^Thr^, suggesting that GABA can mediate excitatory effects already under these conditions.

It is important to note that in the present study we considered only E_GABA_ as the relevant parameter, which in reality depends not only on [Cl^−^]_i_ but also on [HCO_3_^−^]_i_ [6]. We have chosen this approach to (i) ease the computational load, (ii) because the consideration of two independent variables makes the interpretation of the results more complicated, and (iii) because the relative HCO_3_^−^ conductance of GABA_A_ receptors differs between distinct neuronal subpopulations [6,77,78]. Differences in intracellular fixed charges can also slightly influence the relation between [Cl^−^]_i_, E_Cl_ and the GABAergic driving force [79,80]. In addition, we did not consider that functionally relevant somato-dendritic [Cl^−^]_i_ gradients exists in neurons [11,81] and that GABAergic synaptic activity, alone or correlated to glutamatergic inputs, considerably alters E_GABA_ [49,53,75,76,82–84]. All of these properties will complicate the prediction of GABAergic response direction, but for any interpretation of the functional consequences of temporal and spatially dynamic [Cl^−^]_i_ (and [HCO_3_^−^]_i_) gradients, it will be necessary to obtain a major framework to understand how the GABAergic response direction depends on the relation between E_GABA_, E_AP_^Thr^ and spatiotemporal synaptic properties. However, to ease the interpretation of the E_GABA_^Thr^ values, we estimated the corresponding [Cl^-^]_i_ using the Goldman equation and realistic parameters for hippocampal neurons (see materials and methods [49]). Using these parameters an E_GABA_ of −50.5 mV corresponds to a [Cl^-^]_i_ of 14.5 mM, an E_GABA_ of −42.8 mV (i.e. E_AP_^Thr^) to 21.7 mM, the E_GABA_^Thr^ of −44.7 mV observed for simultaneous GABA/AMPA synaptic inputs corresponds to 19.7 mM, and the E_GABA_^Thr^ of −47.5 mV observed for moderate tonic GABA inputs corresponds to a [Cl^-^]_i_ of 17.1 mM.

Previous studies reported for early postnatal cortical plate neurons an E_GABA_ between ca. −40 mV [19,85] and −50 mV [86]. Thus the E_GABA_^Thr^ between −42.8 mV and −50.5 mV, determined in the present study for the distinct conditions, is in the range of the experimentally measured E_GABA_ values, indicating that GABA may indeed mediate excitatory as well as inhibitory effects in the immature neocortex. This suggestion is in line with studies reporting both, excitatory and inhibitory GABAergic effects in the immature brain [48,87]. In contrast, in immature hippocampal neurons an E_GABA_ of ca. −55 mV [88] has been reported, which is clearly below the E_GABA_^Thr^ values determined in our study, suggesting a stable inhibitory GABAergic action in this brain region. However, several reports indicate that GABA can mediate excitation in immature hippocampal neurons [8,46,88], which is obviously in contrast to this suggestion. On the other hand, detailed analysis using minimally invasive recording methods indicate that E_m_ and E_Thr_^AP^ are probably substantially more negative than observed with conventional whole-cell or gramicidin-perforated recording techniques, reporting an E_m_ of −77 mV and an E_Thr_^dVdt^ of −46 mV [44,89]. Thus under physiological conditions both, E_m_ and E_Thr_^AP^ are more negative than the values used for our simulation, supporting that a stable, inward directed GABAergic driving force during the first postnatal week can indeed exist [88]. However, all main findings of our in-silico study can directly be transferred to the more realistic conditions determined in the latter study, by applying a linear shift in the absolute values for E_GABA_^Thr^ and E_AP_^ST^ from the used parameters derived from conventional whole-cell recordings in immature hippocampal slices [49] to the more negative values suggested by Valeeva et al. [44].

Also of note is the observation that the threshold conductance g_GABA_^Thr^ determined for the simulation of only GABAergic synapses is orders of magnitude above physiological values for moderate GABAergic inputs of ca. 1 nS [49,53,54] when E_GABA_ is approaching E_GABA_^Thr^. However, please consider that these conditions require that E_GABA_ was only very slightly above E_GABA_^Thr^ and thus each GABAergic synapse contributed only a negligible depolarizing drive. In fact, from these simulations we could estimate that for an E_GABA_ of ca. 0.5 mV positive to E_AP_^ST^ about 20–30 single synaptic inputs were required for a direct suprathreshold response, which is in the range of the observed number of correlated GABAergic inputs during a GDP, an excitatory transient network event depending on excitatory GABAergic synapses [8,49].

The first major result of this in-silico study was the observation that E_GABA_^Thr^ determined for the GABAergic effect on AMPA-mediated inputs was in many cases considerably negative to E_AP_^Thr^, in contrast to the initial theoretical consideration [35,36]. In our experiment we were also able to provide a mechanistic explanation for this observation. First, by using a current-clamp approach we could replicate that the GABAergic depolarization, when isolated from the GABAergic conductance shift, acted excitatory whenever the peak GABAergic depolarization was positive to the RMP, resulting in an E_GABA_^Thr^ of −50.5 mV. This stringent excitatory effect can be easily explained by the fact that in the absence of conductance changes each depolarization brings E_m_ closer to E_AP_^Thr^. Next, we could demonstrate, by providing AMPA-inputs with a defined advance or delay to the GABAergic inputs, a clear bimodal effect of depolarizing GABA responses. In all cases in which the AMPA inputs preceded the GABA input E_GABA_^Thr^ was close to E_AP_^Thr^ (Fig 4H). Under this condition the AP initiation was under the control of the subsequent GABAergic conductance shift. And under this condition, the GABA_A_ receptor will mediate an inward current, corresponding to a putative excitatory effect, as long as E_GABA_ was positive to E_m_, Thereby, an excitatory effect was induced only if E_GABA_ was above E_AP_^Thr^. However, if the AMPA-mediated inputs occurred after the GABAergic inputs, E_GABA_^Thr^ was systematically shifted to more negative values approximating the RMP of −50.5 mV. This effect can be attributed to the fact that the GABAergic depolarization outlasts the GABAergic conductance shift. Thus, under these conditions the depolarization progressively dominates the effect of GABA, leading to a gradual shift in E_GABA_^Thr^ towards more negative potentials. If the GABAergic conductance can be neglected, each depolarizing shift, i.e. each membrane change depolarized to RMP, contributed to the excitation, leading again to an E_GABA_^Thr^ of −50.5 mV. The impact of the temporal profile of GABAergic conductance change vs. GABAergic depolarization on neuronal excitability has already been experimentally addressed in hypothalamic [40] and neocortical [41] neurons, where within the same neuron the initial phase of a GABA response prevented AP initiation, whereas at later time points of the GABAergic responses AP initiation was facilitated. Despite this clear latency-dependent effect, the reciprocal actions of a depolarization-induced facilitation and a conductance-induced shunting inhibition can also explain why E_GABA_^Thr^ for synaptic inputs was neither at RMP, which would be the case if only the membrane potential shift was relevant, nor at E_Thr_^AP^, which would be the case if E_m_ was only dependent on the actual GABAergic conductance. Note in this respect that the passive E_GABA_^Thr^ of −50.5 mV corresponds to an estimated [Cl^-^]_i_ of 14.4 mM.

In immature neurons, with their slow membrane time constants [63,90], the membrane responses are most probably prone to outlast the membrane conductance for both glutamatergic and GABAergic synaptic inputs. On the other hand, this effect of a prolonged membrane time constant in immature neurons may be partially compensated by the fact, that immature synaptic GABAergic currents show significantly longer decay time constants [63], thereby prolonging the interval in which the shunting inhibitory effects contributes to E_GABA_^Thr^. Another important functional consequence of our results is that the timing between GABAergic and glutamatergic inputs critically determines E_GABA_^Thr^. In this respect classical feedforward as well as recurrent inhibition, with its short latency to excitatory inputs [91], will impose a rather strict inhibition even at depolarizing GABAergic conditions as long as E_GABA_ is maintained below E_Thr_^AP^. Thus this kind of inhibitory control would be rather stable upon activity dependent shifts in E_GABA_ [49,76,82,83,92]. On the other hand, for GABAergic inputs that are not temporally correlated with the excitatory inputs, e.g. during blanket inhibition, it must be considered that E_GABA_^Thr^ can be negative to E_AP_^Thr^, and thus may mediate a less stable inhibition that is more sensitive to ionic plasticity.

The second major result of this in-silico study was the observation, that the spatial relation between GABAergic and AMPA inputs also critically affects E_GABA_^Thr^. As expected, our simulation revealed that the inhibitory effect, as quantified by Δg_AMPA_^Thr^, of proximal GABAergic synapses are stronger than that of distally located ones. The Δg_AMPA_^Thr^ values were substantially larger for AMPA synapses located distally to the GABA synapse, indicating that a GABA input can shunt EPSPs from distal synapses, as suggested from in-vitro and in-silico experiments [41]. For proximally located GABA synapses we could observe that E_GABA_ showed only little dependency on the location of the AMPA-mediated inputs. In these cases, E_GABA_^Rev^ amounted to ca. −46 mV, suggesting that both, shunting and depolarizing effects contribute to the impact of GABA on the excitability. In contrast, we observed for distally located GABA synapses a strong dependency of E_GABA_^Thr^ on the location of AMPA-mediated inputs. For such distal GABA synapse locations a negative E_GABA_^Thr^ close to −50 mV was observed at proximal AMPA synapses, which reflects the fact that with this configuration only the electrotonically propagating GABAergic depolarization has an effective influence with the AMPA-mediated depolarization, while the GABAergic conductance shift acts more locally. For co-localized GABA and AMPA synapses at the distal end of the dendrite E_GABA_^Thr^ approximated E_AP_^Thr^ at ca. −43 mV, indicating that here the effect of GABA was mediated mainly by membrane shunting. Intriguingly the “slope” of E_GABA_^Thr^ was steeper for AMPA synapses in the dendritic segment proximal to the GABA synapse. The slope became shallower for the segment distal from the GABA synapse. This observation indicates that for all AMPA synapses distal to the GABA synapse a substantial fraction of the synaptic currents were shunted by the GABAergic conductance before they can affect AP initiation in the soma. In contrast, for all AMPA synapses located proximal to the GABA synapse the shunting effect was diminished with increasing distance between both synapses, whereas the electrotonically propagating depolarization maintained a more stable excitatory influence and thereby shifted E_GABA_^Thr^ towards the RMP. Thus the results of our experiments suggest an additional mechanism that contribute the putative excitatory GABAergic effect of dendritic GABA inputs [41], in addition to the existence of stable or dynamic somato-dendritic [Cl^−^]_i_ gradients [93,94].

These in-silico observations indicate that perisomatic inhibition, which is the dominant form for the classical feedback and feedforward inhibition mediated by parvalbumin-positive interneurons [95,96], can maintain a stable inhibitory effect regardless of the site of glutamatergic inputs and ionic plasticity. On the other hand, the impact of GABAergic synapses located in the dendritic periphery, e.g. by the hippocampal O-LM interneurons [97] or neocortical Martinotti interneurons [98], will critically depend on the location of the depolarizing GABAergic inputs and can putatively mediate an excitatory impact on AMPA synapses close to the soma at slightly depolarizing E_GABA_.

A specific physiological function has been suggested for the synapses of Chandelier cells on the axon initial segment, as it has been reported that these synapses maintain a depolarizing, putatively inhibitory action [70,71]. However, other reports suggest that GABA receptors in the axon initial segment still mediate an inhibitory effect due to a depolarized shift in the AP threshold [99]. Our results indicate that a GABAergic synapse at the axon initial segment mediated an inhibitory action on somatic glutamatergic inputs as long as E_GABA_ was slightly below the E_AP_^Thr^, whereas E_GABA_^Thr^ was shifted to negative values for glutamatergic inputs located in the dendrite. However, the E_GABA_^Thr^ estimated from these simulations only marginally differ from values obtained with a somatic GABAergic synapse and the same organization of glutamatergic inputs. In summary, these results indicate that the GABA synapse in the axon initial segment does not represent a specific synapse when the dependency between E_GABA_ and excitability control was considered. But independent of this conclusion, the specific properties of [Cl^-^]_i_ homeostasis in the axon initial segment [70,71] as well as the impact of this synapse on the AP threshold [99] will still infer rather specific implications of this synapse in regulating neuronal spike output.

For the present simulation we used in the ball and stick model and the reconstructed dendritic topology only passive dendritic membranes. However, in reality dendrites are equipped with a collection of ion channels that underlie non-linear integration and enable active information processing within this compartment [100,101]. It is generally considered that mainly a supralinear integration occurs in active dendrites [100]. The apparent reduction in the dendritic filtering under this supralinear integration would reduce the slope of the E_GABA_^Thr^ gradient between the proximal and distal end of the dendrite (Fig 5). In the dendrite also anterograde APs can be initiated. In hippocampal pyramidal neurons they are generated at higher stimulus intensities than somatic APs [102] and would thus not interfere with the determination of E_GABA_^Thr^. In other case dendritic APs are evoked at lower stimulation thresholds [103], which will most probably led to the situation that E_GABA_^Thr^ will approximate the E_AP_^Thr^ of the dendritic AP.

In addition, our results indicate that for small to moderate tonic GABAergic conductance E_GABA_^Thr^ was systematically more negative than E_AP_^Thr^, which suggests that even at rather moderate depolarizations tonic GABAergic currents can mediate an excitatory effect. Only at higher g_GABA_^tonic^ the E_GABA_^Thr^ approaches E_AP_^Thr^. The results of this simulation replicate the findings of a previous in-vitro study, that demonstrated excitatory effects of depolarizing tonic GABAergic responses at low conductances, whereas at higher conductances a stable inhibition was imposed [104]. Our results are also in line with the excitatory effects of extrasynaptic GABA_A_ receptors in the immature hippocampus [45]. In our simulations E_GABA_^Thr^ remained stable at about −48.3 mV for g_GABA_^tonic^ smaller than ca. 10^−2^ nS/cm^2^, which is close to the passive membrane conductance g_pas_ of 0.0128 nS/cm^2^. We assume that below this value the shunting effects caused by g_GABA_^tonic^ were negligible to the background conductance g_pas_ and thus did not considerably contribute to the shunting of EPSCs. Only if g_GABA_^tonic^ exceeded g_pas_ a relevant additional inhibitory component was imposed by the GABAergic conductances and thus E_GABA_^Thr^ converged towards E_AP_^Thr^.

The results from the simulation in a neuron with a more realistic dendritic morphology are mainly congruent to the results with the simplified dendritic geometry. At high frequencies, which resulted in a high probability that AMPA inputs occurred during the GABAergic conductance shift, we observed that E_GABA_^Thr^ was close to E_AP_^Thr^. Thus under physiological conditions in desynchronized states [105] or during synchronized activity states in the immature brain [8,49], which are characterized by a high frequency of both GABAergic and glutamatergic inputs, GABA mediates a stable inhibitory effect as long as E_GABA_ was slightly below E_AP_^Thr^. As a consequence, such kind of GABAergic inhibition is less prone to activity-dependent [Cl^-^]_i_ increases [76,82,106]. For low frequencies of unsynchronized inputs, which resulted in a high probability that the AMPA inputs happen during the late, depolarization-dominated phase of a GABA response, E_GABA_^Thr^ is less positive, indicating that under such conditions excitatory GABAergic effects can happen at lower [Cl^-^]_i_. In addition, our in-silico experiments with the more realistic dendritic morphology also indicates that proximally located GABAergic synapses, which represents an important class of GABA synapses mediating feedforward inhibition [68], are even more resistant to [Cl^-^]_i_ alterations with an E_GABA_^Thr^ around E_AP_^Thr^. Thus, this type of synapse, located in a dendritic compartment that due to its dimensions is already less prone to dynamic [Cl^-^]_i_ changes, can maintain an inhibitory action already at rather high synaptic activity levels. On the other hand, our simulations also revealed that for uncorrelated GABA and AMPA inputs in a frequency range between 1 and 20 Hz E_GABA_^Thr^ was under all condition above -46 mV, indicating that a substantially high [Cl^-^]_i_ of about 18.5 mM would be required to mediate an excitatory effect. Similar conclusion could be drawn for the influence of tonic GABAergic receptors, where the E_GABA_^Thr^ were at about -45 mV, corresponding to a [Cl^-^]_i_ of about 19.5 mM. We assume that this discrepancy in the E_GABA_^Thr^ values between the reduced and the realistic dendritic model may be caused by the fact that in the approach we used for the model with a simple dendritic morphology (only a single AMPA synapse), we did not consider the effect of tonic GABAergic conductances on the temporal summation of glutamatergic postsynaptic potentials. In summary, the experiments with the more realistic topology indicate that an effect of spatial and temporal relation between AMPA and GABA inputs on E_GABA_^Thr^ exists, but is in the range of few mV. However, from our experiments with random inputs it cannot be excluded that for specific conditions, with remote and temporally separated synaptic inputs, E_GABA_^Thr^ may also be substantially more hyperpolarized.

Another conclusion that could be drawn from our study is that some attention should be taken to the method used to detect the AP threshold. Obviously there is, despite the frequent use of this descriptive parameter, no consensus on the definition of AP threshold [43]. Therefore, we used in this in-silico study four different, established methods for E_AP_^Thr^ detection. Our in-silico experiments demonstrated that the AP threshold value determined from a fixed threshold of dV/dt [44,51], from the first positive peak in d^3^V/dt^3^ [52], and from linear regression of the AP upstroke [37] were comparable at potentials of ca. −34 mV to ca. −38 mV. In contrast, substantially negative values of −42.8 mV were determined if E_AP_^Thr^ was defined as the maximal potential that did not result in AP triggering (E_Thr_^ST^). The difference in the results of these methods can be easily explained by the fact that E_Thr_^ST^ represents a quasi-stationary value (dV/dt close to 0) that is just insufficient to trigger the entry to the Hodgkin cycle. On the other hand, the other three E_AP_^Thr^ values represent distinct states during the dynamic events in the initial AP phase. The fact that in our simulations E_GABA_^Thr^ for only GABAergic inputs indeed approximated E_Thr_^ST^ can be related to the fact that the excitation threshold for GABAergic inputs was also determined under quasi-stationary conditions. For the influence of GABA on synaptic AMPA-mediated inputs the excitation threshold was determined in the interval between the onset of the GABA inputs and the duration at which 63% of the peak depolarization was obtained. Thus, for the relevant traces that distinguished between subthreshold and suprathreshold AMPA inputs, dV/dt was considerable small and thus the AP threshold was also determined under quasi stationary conditions. Under physiological conditions random fluctuation in E_m_ will most probably limit the difference between E_Thr_^dVdt^, E_Thr_^d3^_,_ E_Thr_^IS^, and E_Thr_^ST^. In any way, while addition of membrane noise to the in-silico models and/or a different methodological definition of the excitation threshold for GABA- and AMPA-mediated inputs would probably change the absolute values for E_GABA_^Thr^ and E_AP_^Thr^, it would not substantially interfere with the main observation of this study, that E_GABA_^Thr^ is for many physiologically relevant situations negative to E_AP_^Thr^.

In conclusion, this simulation indicates that, in addition to the influence of short-term and long-term ionic plasticity, the uneven distribution of [Cl^−^]_i_ gradients within individual cells and the effects of tonic and phasic inhibition [10,11,76,82], the observed spatial and temporal constraints on the E_GABA_ to E_AP_^Thr^ relation imposes another level of complexity to the dynamic properties of GABAergic inhibition/excitation. While on one hand our results support the textbook knowledge that GABA mediates a stable inhibition as long as hyperpolarizing membrane responses are evoked (or [Cl^−^]_i_ is sufficiently low), on the other hand the altered [Cl^−^]_i_ homeostasis in early development and several neurological conditions like trauma, stroke or epilepsy [11,12,30,31], can impact the level of inhibitory control already upon moderate [Cl^−^]_i_ changes in a complex way.

## 4. Materials and methods

### 4.1 Ethics statement

All experiments were conducted in accordance with EU directive 86/609/EEC for the use of animals in research and the NIH Guide for the Care and Use of Laboratory Animals, and were approved by the local ethical committee (Landesuntersuchungsanstalt RLP, Koblenz, Germany). We made all efforts to minimize the number of animals and their suffering.

### 4.2. Electrophysiological procedures

#### 4.2.1. Slice preparation

Newborn pups of postnatal days [P] 4–7 were obtained from time pregnant C57Bl/6 mice (Janvier Labs, Saint Berthevin, France) housed in the local animal facility at 12/12 day/night cycle and ad libitum access to food and water. The mouse pups were decapitated in deep enflurane (Ethrane, Abbot Laboratories, Wiesbaden, Germany) anaesthesia, their brains were quickly removed and immersed for 2–3 min in ice-cold standard artificial cerebrospinal fluid (ACSF, 125 mM NaCl, 25 mM NaHCO_3_, 1.25 mM NaH_2_PO_5_, 1 mM MgCl_2_, 2 mM CaCl_2_, 2.5 mM KCl, 10 mM glucose, equilibrated with 95% O_2_ / 5% CO_2_, osmolarity 306 mOsm). Four hundred μm thick horizontal slices including the hippocampus were cut on a vibratome (Microm HM 650 V, Thermo Fischer Scientific, Schwerte, Germany) and subsequently stored in an incubation chamber filled with oxygenated ACSF at room temperature for at least 1h before they were transferred to the recording chamber.

#### 4.2.2 Patch-clamp recordings

Whole-cell patch-clamp recordings were performed at 31 ± 1°C in a submerged-type recording chamber attached to the fixed stage of a microscope (BX51 WI, Olympus). Pyramidal neurons in the stratum pyramidale of the CA3 region were identified by their location and morphological appearance in infrared differential interference contrast image. Patch-pipettes (5–12 MΩ) were pulled from borosilicate glass capillaries (2.0 mm outside, 1.16 mm inside diameter, Science Products, Hofheim, Germany) on a vertical puller (PP-830, Narishige) and filled with the pipette solutions (86 mM K-gluconate, 44 mM KCl, 4 mM NaCl, 1 mM CaCl_2_, 11 mM EGTA, 10 mM K-HEPES, 2 mM Mg2-ATP, 0.5 mM Na-GTP, pH adjusted to 7.4 with KOH and osmolarity to 306 mOsm with sucrose). In few experiments 40 mM KCl were replaced with 40 mM K-gluconate. Signals were recorded with a discontinuous voltage-clamp/current-clamp amplifier (SEC05L, NPI, Tamm, Germany), low-pass filtered at 3 kHz and stored and analyzed using an ITC-1600 AD/DA board (HEKA) and TIDA software. All voltages were corrected post-hoc for liquid junction potentials of -8 mV for a pipette [Cl^−^] of 10 mM and -5 mV for 50 mM [20]. Input resistance and capacitance were determined from a series of hyperpolarizing current steps. Action potentials (AP) were induced by a series of depolarizing current steps. For averaging of AP wave forms the first AP from traces that showed a series of APs were used.

### 4.3. Compartmental modeling

The compartmental modeling was performed using the NEURON environment (neuron.yale.edu). The source code of all models and stimulation files used in the present paper can be found in ModelDB (http://modeldb.yale.edu/267142). For compartmental modelling we used either a simple ball (soma diameter = 43 μm) or a ball and stick model (soma with d = 43 μm, linear dendrite with L = 1000 μm, diameter 1 μm, and 301 segments). In both models a passive conductance (g_pas_) with a density of 1.28*10^−5^ nS/cm^2^ and a reversal potential (E_pas_) of −50.5 mV was distributed, which resulted for the ball-and-stick model in passive membrane properties that were comparable to the properties of recorded pyramidal CA3 neurons. Active membrane properties were in the majority of the experiments incorporated only in the somatic compartment. In one experiment we added to the ball and stick model an axon containing one initial segment (L = 10 μm, diameter = 0.2 μm) with active AP properties and remaining 10 segments (L = 10 μm, diameter = 0.2 μm) in which the Na^+^ and K^+^ peak conductivity was reduced by 10% [99]. In this experiment the GABA synapse was restricted to the axon initial segment.

Because it was not possible to generate a reasonable sharp AP onset with a standard Hodgkin-Huxley (HH) model and since we are particularly interested in the AP threshold properties, we adapted a model developed by Naundorf et al. [50]. This model considered three different states for the Na^+^ channels:

$$Na_{o}=open\;state$$

$$Na_{c}=closed\;state$$

$$Na_{i}=inactivated\;state$$

With mutual transitions between Na_o_ and Na_c_ as well as Na_c_ and Na_i_ and a mono-directional transition from Na_o_ to Na_i._ The rate functions α_A_(V) for the transition Na_c_➙Na_o_ and α_IC_(V) for the transition Na_i_➙Na_c_ are described by the functions:

$$\alpha_{A}(V_{t})=\frac{Q_{10}}{\tau_{Naact}}\times \frac{G_{c\to o}^{Na}}{\left(1+e^{\left(\frac{\left(V_{c\to o}^{Na}-V_{t}\right)}{k_{c\to o}^{Na}}\right)}\right)}and\;\alpha_{IC}(V_{t})=\frac{Q_{10}}{\tau_{Naina}}\times \frac{G_{i\to c}^{Na}}{\left(1+e^{\left(\frac{\left(V_{i\to c}^{Na}-V_{t}\right)}{k_{i\to c}^{Na}}\right)}\right)}.$$

The rate functions β_A_(V) for the transition Na_c_➙Na_o_ and β_IC_(V) for the transition Na_c_➙Na_i_ are described by the function:

$$\beta_{A}(V_{t})=\frac{Q_{10}}{\tau_{Naact}}\times \frac{G_{o\to c}^{Na}}{\left(1+e^{\left(\frac{\left(V_{t}-V_{c\to o}^{Na}\right)}{k_{c\to o}^{Na}}\right)}\right)}and\;\beta_{IC}(V_{t})=\frac{Q_{10}}{\tau_{Naina}}\times \frac{G_{i\to c}^{Na}}{\left(1+e^{\left(\frac{\left(V_{t}-V_{c\to c}^{Na}\right)}{k_{i\to c}^{Ni}}\right)}\right)}.$$

The voltage independent relaxation from *Na*_*o*_ occurs with the rate constant τ_Na_.

The dynamic properties of the fraction of open Na^+^ channels O_Na_ and inactivated Na^+^ channels H_Na_ were described by the differential equations:

$${\dot{O}}_{Na}=\alpha_{A}(v+{cf}_{Na}O_{Na})(1-O_{Na}-H_{Na})-\beta_{A}(v+{cf}_{Na}O_{Na})O_{Na}-\frac{O_{Na}}{\tau_{Na}}$$

$${\dot{H}}_{Na}=\alpha_{IC}(v)(1-H_{Na})-\beta_{IC}(v)H_{Na}-\frac{H_{Na}}{\tau_{Na}}$$

The cooparativity factor cf_Na_ was introduced by Naudorf et al. to account for the cooperative opening of Na^+^ channels under realistic condition [50].

The actual Na^+^ conductance g_Na_ was given by the equation g_Na_ = g_Na_^Max^ O_Na_.

The Na^+^ current I_Na_ was calculated from g_Na_ and the sodium equilibrium potential e_Na_ according to Ohm’s law:

$$I_{Na}=g_{Na}(v‐e_{Na}).$$

In addition to the exclusive Na^+^ current model published by Naundorf et al., we implemented a simple two state model for the delayed rectifier K^+^ current to enable the simulation of action potentials. The K_c_➙K_o_ transition rate described by the equation:

$$\alpha_{A}^{K}(V_{t})=\frac{Q_{10}}{\tau_{Kact}}\times \frac{G_{c\to o}^{K}}{\left(1+e^{\left(\frac{\left(V_{c\to o}^{K}-V_{t}\right)}{k_{c\to o}^{K}}\right)}\right)}.$$

The K_o_➙K_c_ transition rate was described by the function

$$\beta_{A}^{K}(V_{t})=\frac{Q_{10}}{\tau_{Kina}}\times \frac{G_{i\to c}^{K}}{\left(1+e^{\left(\frac{\left(V_{t}-V_{i\to c}^{K}\right)}{k_{i\to c}^{K}}\right)}\right)}.$$

In addition, a voltage independent relaxation from K_*o*_ with the rate constant τ_K_. was considered.

The dynamic properties of the open fraction of K^+^ channels (O_K_) was described by the differential equations using the cooperativity factor cf_K_:

$${\dot{O}}_{K}=\alpha_{A}(v+{cf}_{K}O_{Na})(1-O_{K})-\beta_{A}(v+{cf}_{K}O_{K})O_{K}-\frac{O_{K}}{\tau_{K}}.$$

The actual K^+^ conductance g_K_ was given by the equation g_K_ = g_K_^Max^O_K_ and the K^+^ current (I_K)_ was calculated by the equation I_K_ = g_K_(v-e_K_).

All parameters were optimized by stepwise approximation to obtain a sufficient fit to the average experimentally determined AP trace, which was quantified by minimizing the root of the summarized squared errors according to the following error weight function:

$$Error=10\times \sqrt{{\left(E_{Thr}^{d3}\right)}^{2}}+3\times \sqrt{{\left(v_{rise}^{max}\right)}^{2}}+\sqrt{{\left(v_{decay}^{max}\right)}^{2}}+\sqrt{{\left(d_{1/2}\right)}^{2}}+\sqrt{{\left(E_{AP}^{Peak}\right)}^{2}}.$$

This error weight function was used with the rationale to put special emphasis for the fitting routine to the dynamic properties at E_AP_^Thr^. The used parameters are given in S4 Table.

AMPA synapses were modeled by an Exp2Syn point process using a reversal potential of -12 mV, a tau1 value of 0.1 ms and a tau2 value of 11 ms, in accordance with the experimentally determined value [49]. GABA synapses were modeled by an Exp2Syn point process using a tau1 value of 0.1 ms and a tau2 value of 37 ms, in accordance with the experimentally determined value [49]. The reversal potential of the GABAergic synapses was the main variable of interest in these simulations. For tonic GABAergic currents a constant membrane conductance was distributed over all membrane with conductance densities and reversal potentials as given in the results part [53].

For the determination of g_GABA_^Thr^ we used an iterative approach where g_GABA_ was first increased by 1 nS steps until an AP was induced within an interval of 800 ms after the GABA input. Subsequently g_GABA_ was decreased by 0.33 nS steps until the AP vanished, followed again by an increase in g_GABA_ by 0.1 pS until the AP reappeared. This alternating sequence was repeated 6 times using a g_GABA_ of 1/10 for each subsequent round. In these experiments E_AP_^Thr^ was defined as the peak voltage of the last subthreshold sweep.

A similar approach was also used to determine g_AMPA_^Thr^. Here g_AMPA_ was initially increased by 0.01 nS steps until an AP was induced. The analysis interval was in all sweeps set to the interval between stimulus onset and the time point when the AMPA-mediated depolarization, determined in the absence of an AP mechanism, decreased to 63% of the peak amplitude. Subsequent g_AMPA_ was decreased by 3.3 pS until the AP disappears, followed by 6 rounds of alternating increasing/decreasing g_AMPA_ steps, with g_AMPA_ step values decreasing by 1/10 for each round.

Due to this iterative approach 55 ± 2.2 sweeps (in n = 22 simulations) were required for each of the 1827 parameters tested to determine g_GABA_^Thr^ and 63.3 ± 0.8 sweeps (in n = 85 simulations) were required for each of the 29730 parameters tested to determine E_GABA_^Thr^. In consequence, between 37348 (Fig 4C) and 534907 (Fig 5B) sweeps are required to test a hypothesis.

To determine E_GABA_^Thr^ under more realistic conditions, we used a dendritic model derived from a reconstructed, biocytin-labeled CA3 pyramidal neuron (Fig 7A and 7B) [49]. This model consists of a soma (diameter 15 μm) and 56 dendrites, that contained between 2 and 193 segments, as adapted from the reconstruction. To determine E_GABA_^Thr^ in this model 2, 10, 20 or 40 GABA and AMPA synapses, for respective stimulation frequencies of 1, 5, 10 or 20 Hz, were randomly distributed across the dendritic compartment and each synapse was stimulated at a random time point during the 2 s stimulation interval. The value for *g*_*AMPA*_ was set to values that corresponds to a spike probability (pAP) of 0.5, determined for each frequency from 999 single sweeps using the same random number sequence in the absence of GABAergic inputs. The p_AP_^50^ was calculated from a linear interpolation of the two values closest to a p_AP_ of 50%. E_GABA_^Thr^ was determined from the E_GABA_ values at which the p_AP_ curve obtained in 999 sweeps reaches the p_AP_^50^ value determined with the same AMPA stimulation pattern in the absence of GABA.

The [Cl^-^]_i_ was estimated from the determined E_GABA_^Thr^ using the Goldman-Hodgkin-Katz equation as follows:

$${\left[{Cl}^{-}\right]}_{i}=10^{\frac{E_{GABA}}{60mV}}({P_{Cl}[{Cl}^{-}]}_{e}+P_{HCO3}{\left[{{HCO}_{3}}^{-}\right]}_{e})-P_{HCO3}{\left[{{HCO}_{3}}^{-}\right]}_{i}$$

using a [Cl^-^]_e_ of 133.5 mM, an extracellular HCO_3_^-^ concentration ([HCO_3_^-^]_e_) of 24 mM, a [HCO_3_^-^]_i_ of 14.1 mM, and a relative HCO_3_^-^ permeability (P_HCO_) of 0.44 [49].

## Supporting information

S1 Fig Characterization of AP properties using different dt values for the simulation. A: Simulated voltage traces using different dt as indicated in the plot. Note the slightly divergent AP shape at 0.05 ms, while at a dt of 0.5 ms oscillations occur. B: Rate of E_m_ changes during an action potential. C: Typical E_AP_^Thr^ values determined with 3 different algorithms on the traces obtained at different dt. Note that all E_Thr_^IS^, E_Thr_^dV/dt^ and E_Thr_^d3^ remained stable for a dt ≤ 0.025 ms.
(TIF)

S2 Fig Effect of a GABA synapse on the excitability of a ball and stick neuron with an added axon. AP mechanisms were restricted to the axon and the GABA synapse was located at the somatic end of the axon (axon initial segment). A: Plot of Δg_AMPA_^Thr^ versus E_GABA_ at different g_GABA_ values for an AMPA synapse located at the soma. B: Plot of E_GABA_^Thr^ at different g_GABA_ for somatic AMPA receptors. Note that E_GABA_^Thr^ was ca. -44.2 mV for physiological g_GABA_ and was shifted towards E_Thr_^IS^ at higher g_GABA_. C: Plot of E_GABA_^Thr^ at different g_GABA_ for dendritic AMPA receptors located at 25%, 50% and 57% of the dendrite, as indicated by the color code. The grey trace represents the somatic AMPA stimulation. Note that E_GABA_^Thr^ was systematically shifted towards lower values with more distant dendritic location.
(TIF)

S1 Table List of g_AMPA_ values required to reach 50% probability for spike initiation at different distributions and frequencies of AMPA inputs. (DOCX)

S2 Table Summary of EGABA^Thr^ values determined in the experiments with the reconstructed dendritic topology using synaptic GABAergic currents. (DOCX)

S3 Table Summary of EGABA^Thr^ values determined in the experiments with the reconstructed dendritic topology using tonic GABAergic currents. (DOCX)

S4 Table Parameters used for the modified Naundorf model. (DOCX)
