# Supplementary figures and images for: Modelling the spatial and temporal constrains of the GABAergic influence on neuronal excitability
Source: PLoS Comput Biol. 2021 Nov 12;17(11):e1009199. doi: 10.1371/journal.pcbi.1009199 (PMC8612559; doi:10.1371/journal.pcbi.1009199)

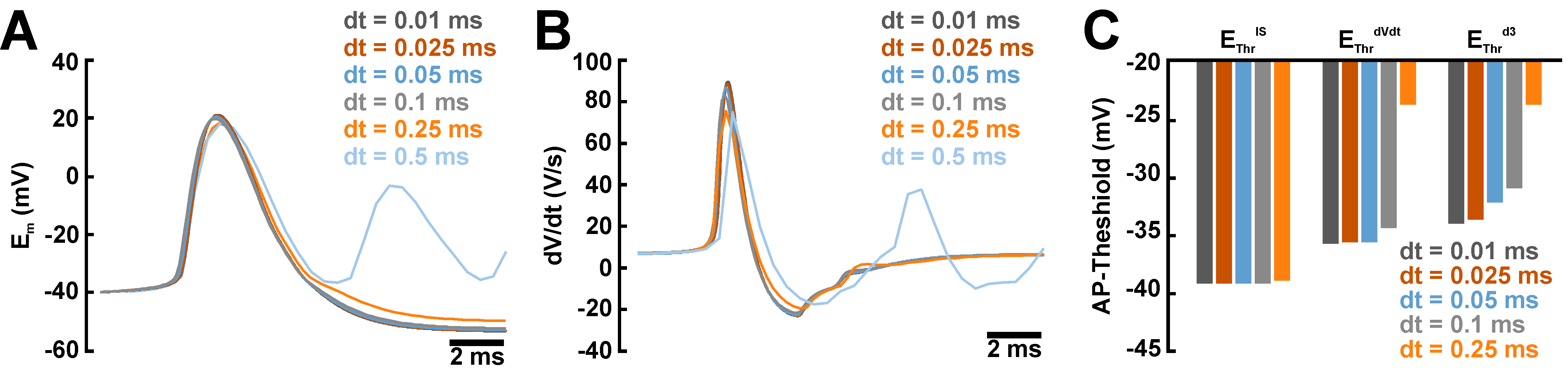

Supplement: S1 Fig — A: Simulated voltage traces using different dt as indicated in the plot. Note the slightly divergent AP shape at 0.05 ms, while at a dt of 0.5 ms oscillations occur. B: Rate of Em changes during an action potential. C: Typical EAPThr values determined with 3 different algorithms on the traces obtained at different dt. Note that all EThrIS, EThrdV/dt and EThrd3 remained stable for a dt ≤ 0.025 ms. (TIF) [file pcbi.1009199.s001.tif]

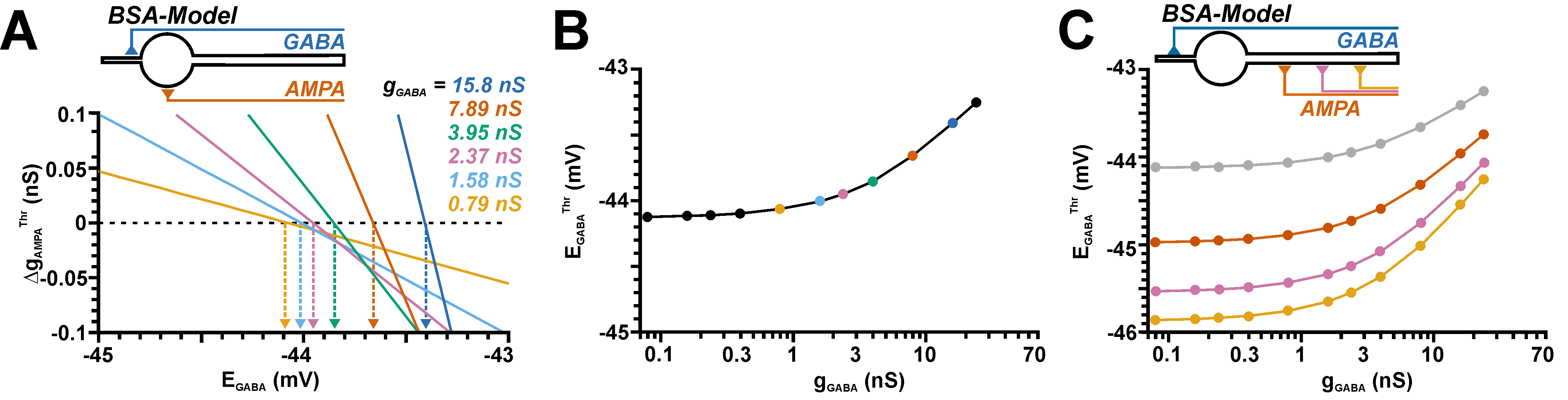

Supplement: S2 Fig — AP mechanisms were restricted to the axon and the GABA synapse was located at the somatic end of the axon (axon initial segment). A: Plot of ΔgAMPAThr versus EGABA at different gGABA values for an AMPA synapse located at the soma. B: Plot of EGABAThr at different gGABA for somatic AMPA receptors. Note that EGABAThr was ca. -44.2 mV for physiological gGABA and was shifted towards EThrIS at higher gGABA. C: Plot of EGABAThr at different gGABA for dendritic AMPA receptors located at 25%, 50% and 57% of the dendrite, as indicated by the color code. The grey trace represents the somatic AMPA stimulation. Note that EGABAThr was systematically shifted towards lower values with more distant dendritic location. (TIF) [file pcbi.1009199.s002.tif]
