# Supplementary material for: Modelling the spatial and temporal constrains of the GABAergic influence on neuronal excitability
Source: PLoS Comput Biol. 2021 Nov 12;17(11):e1009199. doi: 10.1371/journal.pcbi.1009199 (PMC8612559; doi:10.1371/journal.pcbi.1009199)
Supplement: S1 Table — (DOCX) [file pcbi.1009199.s003.docx]

| **gAMPA currents required for a pAP of 0.5** | | | | |
| --- | --- | --- | --- | --- |
|  | **Frequency** | | | |
| **Distribution** | **1 Hz** | **5 Hz** | **10 Hz** | **20 Hz** |
| evenly | 0.920 pS | 0.551 pS | 0.394 pS | 0.275 pS |
| proximal | 0.782 pS | 0.487 pS | 0.359 pS | 0.253 pS |
| distal | 1.078 pS | 0.632 pS | 0.445 pS | 0.302 pS |
