# Supplementary material for: Modelling the spatial and temporal constrains of the GABAergic influence on neuronal excitability
Source: PLoS Comput Biol. 2021 Nov 12;17(11):e1009199. doi: 10.1371/journal.pcbi.1009199 (PMC8612559; doi:10.1371/journal.pcbi.1009199)
Supplement: S2 Table — (DOCX) [file pcbi.1009199.s004.docx]

| **Phasic GABA - evenly distrib. GABA inputs - evenly distrib. AMPA inputs** | | | | |
| --- | --- | --- | --- | --- |
| **g_GABA_** | **1 Hz** | **5 Hz** | **10 Hz** | **20 Hz** |
| 0.789 pS | -45.88 mV | -45.27 mV | -44.01 mV | -43.50 mV |
| 2.367 pS | -45.64 mV | -44.73 mV | -43.49 mV | -43.01 mV |
| 7.89 pS | -45.88 mV | -44.11 mV | -42.94 mV | -42.58 mV |
| **Phasic GABA - proximal GABA inputs - evenly distrib. AMPA inputs** | | | | |
| **g_GABA_** | **1 Hz** | **5 Hz** | **10 Hz** | **20 Hz** |
| 0.789 pS | -45.70 mV | -44.98 mV | -43.87 mV | -43.41 mV |
| 2.367 pS | -44.90 mV | -44.26 mV | -43.22 mV | -42.82 mV |
| 7.89 pS | -45.70 mV | -43.53 mV | -42.60 mV | -42.06 mV |
| **Phasic GABA - distal GABA inputs - evenly distrib. AMPA inputs** | | | | |
| **g_GABA_** | **1 Hz** | **5 Hz** | **10 Hz** | **20 Hz** |
| 0.789 pS | -45.83 mV | -45.48 mV | -44.16 mV | -43.62 mV |
| 2.367 pS | -45.83 mV | -45.14 mV | -43.72 mV | -43.35 mV |
| 7.89 pS | -45.83 mV | -44.64 mV | -43.44 mV | -42.94 mV |
| **Phasic GABA - proximal GABA inputs - distal AMPA inputs** | | | | |
| **g_GABA_** | **1 Hz** | **5 Hz** | **10 Hz** | **20 Hz** |
| 0.789 pS | -45.69 mV | -44.07 mV | -43.88 mV | -43.32 mV |
| 2.367 pS | -45.21 mV | -43.81 mV | -43.21 mV | -42.72 mV |
| 7.89 pS | -45.69 mV | -43.14 mV | -42.57 mV | -41.95 mV |
| **Phasic GABA - distal GABA inputs - proximal AMPA inputs** | | | | |
| **g_GABA_** | **1 Hz** | **5 Hz** | **10 Hz** | **20 Hz** |
| 0.789 pS | -45.62 mV | -45.42 mV | -45.19 mV | -44.58 mV |
| 2.367 pS | -45.72 mV | -45.33 mV | -44.79 mV | -44.25 mV |
| 7.89 pS | -45.62 mV | -45.15 mV | -44.46 mV | -43.86 mV |
