# Supplementary material for: Modelling the spatial and temporal constrains of the GABAergic influence on neuronal excitability
Source: PLoS Comput Biol. 2021 Nov 12;17(11):e1009199. doi: 10.1371/journal.pcbi.1009199 (PMC8612559; doi:10.1371/journal.pcbi.1009199)
Supplement: S3 Table — (DOCX) [file pcbi.1009199.s005.docx]

| **tonic GABA - evenly distributed AMPA inputs** | | | | |
| --- | --- | --- | --- | --- |
| **g_GABA_** | **1 Hz** | **5 Hz** | **10 Hz** | **20 Hz** |
| 0.875 pS/cm^2^ | -44 mV | -45.33 mV | -44 mV | -43.75 mV |
| 1.75 pS/cm^2^ | -44.6 mV | -44.92 mV | -44.17 mV | -43.78 mV |
| 4.375 pS/cm^2^ | -44.95 mV | -45.08 mV | -44.39 mV | -43.83 mV |
| 8.75 pS/cm^2^ | -44.98 mV | -44.88 mV | -44.33 mV | -43.85 mV |
| 17.5 pS/cm^2^ | -44.86 mV | -44.61 mV | -44.04 mV | -43.74 mV |
| 43.75 pS/cm^2^ | -44.31 mV | -44.01 mV | -43.62 mV | -43.27 mV |
| 87.5 pS/cm^2^ | -43.53 mV | -43.41 mV | -42.99 mV | -42.76 mV |
