# Supplementary material for: Modelling the spatial and temporal constrains of the GABAergic influence on neuronal excitability
Source: PLoS Comput Biol. 2021 Nov 12;17(11):e1009199. doi: 10.1371/journal.pcbi.1009199 (PMC8612559; doi:10.1371/journal.pcbi.1009199)
Supplement: S4 Table — (DOCX) [file pcbi.1009199.s006.docx]

| Parameter | Value | Description |
| --- | --- | --- |
| **Na^+^ Channels** | | |
| τ^Na^_act_ | 0.51 | Time constant describing the Na_c_➙Na_o_ transition |
| V^Na^_c➙o_ | -32.8 mV | V_1/2_ for Bolzmann-Function describing the Na_c_➙Na_o_ transition |
| k^Na^_c➙o_ | 3 | Slope for Bolzmann-Function describing the Na_c_➙Na_o_ transition |
| τ^Na^_ina_ | 1.1 | Time constant describing the Na_i_➙Na_c_ transition |
| V^Na^_i➙c_ | -35.7 mV | V_1/2_ for Bolzmann-Function describing the Na_i_➙Na_c_ transition |
| k^Na^_i➙c_ | 5 | Slope for Bolzmann-Function describing the Na_i_➙Na_c_ transition |
| τ_Na_ | 999 | Rate constant for voltage independent relaxation of Na^+^ channels |
| cf_Na_ | 14 | Cooperativity factor for Na^+^ channel opening |
| g_Na_^Max^ | 0.006 (S/cm^2^) | Conductance density for Na^+^ channels in the soma |
| E_Na_ | 66 (mV) | Reversal potential for Na^+^ currents |
|  |  |  |
| **K^+^ Channels** | | |
| τ^K^_act_ | 1.5 | Time constant describing describing the K_c_➙K_o_ transition |
| V^K^_c➙o_ | -30 mV | V_1/2_ for Bolzmann-Function describing the K_c_➙K_o_ transition |
| k^K^_c➙o_ | 4 | Slope for Bolzmann-Function describing the K_c_➙K_o_ transition |
| τ^K^_ina_ | 1.66 | Time constant describing describing the K_o_➙K_i_ transition |
| V^K^_o➙c_ | -10 mV | V_1/2_ for Bolzmann-Function describing the K_o_➙K_i_ transition |
| k^K^_o➙c_ | 1 | Slope for Bolzmann-Function describing the K_o_➙K_i_ transition |
| τ_K_ | 500 | Rate constant for voltage independent relaxation of K^+^ channels |
| cf_K_ | 0 | Cooperativity factor for K^+^ channel opening |
| g_K_ | 0.0006 (S/cm^2^) | Conductance density for K^+^ channels in the soma |
| E_K_ | -84 (mV) | Reversal potential for K^+^ currents |
