# Supplementary material for: Modelling the spatial and temporal constrains of the GABAergic influence on neuronal excitability
Source: PLoS Comput Biol. 2021 Nov 12;17(11):e1009199. doi: 10.1371/journal.pcbi.1009199 (PMC8612559; doi:10.1371/journal.pcbi.1009199)
Supplement: Attachment — Submitted filename: Lombardi et al Spatial_Rheobase_Reviewer Comments_Final.pdf [file pcbi.1009199.s007.pdf]

Dear Editor,

Thank you very much for giving us the opportunity to response to the reviewer's excellent concerns and to optimize the present manuscript in accordance to their helpful suggestions. All changes on the manuscript are redlined in the reviewers vesion of the manuscript. Please find our detailed responses to the reviewers' comments in the following.

Reviewer #1:

This paper uses computational methods to thoroughly answer a simple but very important question. At what Egaba values and under which conditions is GABAaR mediated synaptic signalling excitatory (ie drive AP generation) versus inhibitory (reduce the likelihood of AP generation)? I am very enthusiastic about this paper and think it is an important contribution to our understanding of GABAergic synaptic transmission. I must say I haven't felt this satisfied after reading a paper for quite a while. It is elegantly simple, and very satisfying in its conclusions. The major conceptual advance is that the authors come up with a metric 'EgabaThr', which defines the Egaba value at which GABAaR mediated synaptic transmission mediates excitation. They then use this to show some intuitive, but important results including the fact that EgabaThr is often actually negative of the action potential threshold. This is particularly the case for two conditions which they demonstrate beautifully: 1) when GABAaR synapses come before AMPA inputs in time, or 2) when GABAaR inputs are more distal to inputs AMPA inputs in space. They also demonstrate that for low conductance tonic GABAaR, EgabaThr is more negative than the action potential threshold. These are all important insights and the field is richer for them. The simulations are appropriate and well performed, the manuscript is well written and logically developed I therefore endorse its publication.

Thank you very much for the careful and kind evaluation.

I only have two minor comments:

1a) Although I presume this was the case, can the authors confirm that active mechanisms (ie voltage gated Na+ and K+) only in the somatic compartment and that the dendrite is passive, and make this explicit in the methods.

The reviewer is right, we used always a passive dendrite and restricted the active mechanisms to the soma (note that for one experiment in the revised manuscript we also included an axon with active properties). To follow the reviewer's concern, we now explicitly stated that the active properties were incorporated only in the soma and for one experiment in the axon (line 773ff).

1b) Further when authors explore the effects of varying the spatial position of the GABAaR synapse on the dendrite the authors are determining the effect on excitability as measured by AP generation at the soma. In the discussion it may be worth the authors acknowledging this somatocentric viewpoint of GABAergic inhibition especially as in many types of neurons (including the CA3 pyramidal neurons modelled here) that dendrites host active conductances and that non-linear input summation and integration also occurs within the dendritic tree itself. This was not modelled here and this should be acknowledged as it is likely that an effect of distally targeted dendritic GABA is to control local active conductances in the dendrite itself (a dendrocentric viewpoint), which could complicate the picture a bit. I'm not suggesting the authors model active dendritic conductances but if they could perhaps acknoweledge this point in the discussion that would be helpful.

We thank the reviewer for this suggestion. We initially considered to simulate active dendritic properties for the revision of this study, but came to the conclusions that the influence of GABA inputs at different E_{GABA} on the various conductances that underlie the active properties of dendritic computation (e.g. Na_v, Ca_v, or HCN channels) is by far too complex to be covered within the scope of the present manuscript. However, we included a short discussion whether active dendritic properties can interfere with the effect of E_{GABA} in the revised manuscript (line 693).

2) I believe in Line 440 " gGABAThr " should be "gampaThr"

We corrected this statement (now in line 1255).

Reviewer #2:

The authors investigate the value of EGABA at which a GABA synapse triggers an action potential in a simple in silico models. The authors investigate the important relations between this threshold value, the GABA conductance, the presence of an AMPA synapse, the tonic GABA conductance and the distance between the GABA synapse and the soma. They found importantly that there are complex relations between the GABA threshold value and all of these parameters.

OVERALL APPRECIATION: The paper is well written and the methodology is sound. The authors share their code which seems to me to be ok. The questions tackled in this work are important and of interest. However, it is difficult for me to appreciate to biological implications of this work due in part to the rather simple nature of the model. For example, the GABA conductances mentioned in the paper are sometimes order of magnitudes larger than realistic values for GABA synapses. As there is a lot of

variability from cell to cell and as membrane potential fluctuates in time, it is not clear to what extent it is relevant to give a precise value for the threshold value of EGABA. What would be the results if the authors added an axon in the model and assumed that the GABA synapse is in the axon initial segment? What would be the results in a more realistic scenarios of several randomly distributed synapses?

Thank you very much for your kind appreciation and your valuable suggestions. In general, we held our models a simple as possible (even using the completely non-physiological ball model) to comprehend the main basic principles underlying the interaction between depolarizing GABAergic responses and the excitability of the membrane. Of course, we are also highly interested in the question, how our observations would translate in a more realistic scenario. However, a thorough evaluation of the impact of a more complex dendritic topology on the interplay between GABA and glutamatergic inputs would require a lot of additional independent parameters. But to follow your suggestion we included for the revision of the manuscript a dendritic model with a realistic topology, derived for a CA3 neuron in the immature mouse hippocampus Lombardi et al., 2018). With this model we investigated at different E_{GABA} values how different rates of random synaptic GABAergic inputs as well as tonic GABAergic currents influence the overall excitability (monitored by the threshold g_{AMPA} values required to trigger APs). Please find the results of these additional experiments described in line 391-456, discussed in line 664-693, and illustrated in Fig. 7-10.

Regarding your question what results would be expected if an axon with a GABAergic input at the axon initial segment was added to the model, we also implemented this topology and calculated the threshold E_{GABA} value for this model. Please find these results described in line 342-356, discussed in line 625-638, and illustrated in the Suppl. Fig. 2.

Regarding you question what result would be expected using a scenario with several randomly distributed synapses, we implemented, as mentioned before, such synapses in the more realistic dendritic scenario.

Specific comments:
1) In figure 1, The action potentials in A and B look rather different with the simulated train of AP exhibiting after spike hyperpolarization which seems to be absent in the recorded trace. How to explain this difference?

This obvious difference was caused by the facts that we (i) neglected the amount of hyperpolarization when picking a “typical” recorded trace and (ii) that we optimized the Markow parameters to fit the rising/decaying phase of the AP. To follow the reviewer’s concern, we (i) now picked a recorded trace that also was in the Mean ± SD range for the peak hyperpolarization (Fig. 1A) and (ii) generated simulated action potentials with a slightly less pronounced hyperpolarization (Fig.

1B-E), due to the fact that we now used another mechanism to simulate the APs (see comment 7). However, we like to state that, because the AP onset in the only relevant AP parameter for our study, we (i) concentrated mainly on the AP threshold, AP rising phase and duration when optimizing the parameters and (ii) like to emphasize that the amount and/or kinetics of the hyperpolarization has only a minor impact of the results of our study (relevant only for the random stimulation at high frequecies).

2) Line 174. The authors mention 'several hundred sweeps'. Could they explain a bit more? Is it because of the Markov modelling of the spike generating channels or to sweep accross all of the parameter values? What is the impact of the number of sweeps and how did they author determined this value?

Due to our algorithm that determined the threshold g_{GABA} or g_{AMPA} values by an alternating stepwise increase and decrease patterns in these values (please see Fig. 2A for an illustration), the exact number of the simulation sweeps required to detect the threshold values is variable for each analyzed parameter set. For the previous version of the manuscript we did not record the exact number of sweeps, we just roughly estimated how much of the alternate sweeps were required to determine the threshold g_{GABA}. To follow the reviewer's concern, and because we are recalculating all simulations again anyway (see comment 7), we now implemented a "sweep counter" in our algorithms and provided the exact number of sweeps in the results part (line 170-173) and the Materials & Methods (line 840-843) of the revised manuscript.

3) In fig 2, you have conductance values in several hundreds of nS, this range seems exagerated to me as gaba synaptic conductance should be in the order of 1nS.

The GABAergic conductances required to directly trigger an AP close to E_{GABA}^{Thr} are indeed really high, as already mentioned in the previous and revised version of the manuscript (line 243-245). However, we are not concerned about this observation, because in this experiment we artificially approach the threshold E_{GABA} value, which is defined as the E_{GABA} at which even the highest g_{GABA} is just insufficient to trigger an AP. When E_{GABA} was about 0.5 mV positive to E_{GABA}^{Thr} amounted to ca. 20 nS, corresponding to ca. 25 single synaptic inputs (with a g_{GABA} of 0.789 nS). This is in the range of the estimated 101 GABAergic inputs that underlie a GDP (Lombardi et al., 2018), as a physiological example of an excitatory GABA driven event. Thus we conclude from our results, that single GABAergic inputs can under most conditions not trigger suprathreshold responses (as explicitly mentioned in the manuscript in line 243-245). However, to follow the reviewer's concern, we now discuss this issue in more detail in the revised version of the manuscript (line 533-541).

4) Did you try any topology beyond the ball and stick one? What do you believe would be the impact of a more complex topology?

In order to follow the reviewer's suggestion, we implemented our algorithms also to a more realistic neuronal topology. For this purpose, we used a reconstructed dendritic topology from an immature hippocampal CA3 neurons, that we already used for previous publications (Lombardi et al 2018, 2019). Please see our comments on this suggestion to the general evaluation.

5) In fig 3 E and F, I think we see the limit of the numerical precision as the lines seem quite irregular especially in 3 F. Is the 'noise' indeed due to numerical limitations or is there something else at play, please discuss.

The authors is right, the "noise" in Fig. 3F, but also the "irregularities" in the plots of e.g. Fig. 3D, Fig. 7E, or Fig. 8D are reflecting small irregularities that are observed at the detection limits of the algorithms, due to the fact that we can, of course, only use a limited amount of sweeps per parameter. Thus we only define a range of the limits (]Value inducing no AP; Value inducing AP]). However, please note that the "noise" in the AP threshold displayed in Fig. 3F is in the range of 0.02 mV, and thus probably much smaller that observable in real neurons with their noise levels.

6) I find it difficult to distinguish between the different shades of blue and orange in figure 4. I suggest using a color scheme for which it is easier to distinguish between the different colors. The same goes for figure 5 B.

In order to follow the suggestions made by PLoS for inclusive imaging, we used in the previous version of the manuscript the color scheme "Colorblind 10". However, to follow the reviewer's concern and to make the AMPA traces differ in more than just the shading, we now use another colorblind palette, developed by Bang Wong (2011), for all figures.

7) I find it difficult to understand why the Hodgkin-Huxley is not suitable here? Could you discuss and justify more your choice for the model of voltage gated Na+ and K+ channels?

When starting this project, we assumed that we need a clearly detectable AP theshold, and thus a sharp AP onset, to clearly relate the excitation/inhibition

threshold in E_{GABA} to the AP threshold. Therefore, we strived to use a more suitable AP model than the classical HH model. However, as our results demonstrate that E_{AP}^{ST} rather that other definitions of the AP threshold was related to the level of E_{GABA} at excitation threshold, this question is of less relevance in the revised manuscript. In any way, following this comment and the second concern of reviewer #3, we came to the conclusion that we will no longer use the not jet validated “modified Markov model” for the present study. Following the suggestion of reviewer #3, we instead used the AP model provided by Naundorf et al (2011) for all simulations included in the present manuscript. Please note this change in the AP mechanisms led only to marginal quantitative changes in the results and had no effect on the general conclusions of the manuscript. The use of the model was introduced in line 150 and the details were explained in the methods section (line 780-814). The used parameters were provided in the Suppl. Table 4, and all experimental results, including all figures, were updated accordingly.

8) A conductance ohmic equation is used to compute the current through Na+ and K+ channels. Is that the same for the Cl current? If so, why not use the GHK flux equation which explicitly takes the intracellular and extracellular concentrations of Cl- and HCO3- into account? The results might not be exactly the same and it could be more relevant to relate the investigations to Cl- concentrations instead of EGABA.

As we used in the present manuscript a fixed E_{GABA} (which can be related to a fixed $[Cl^-]_i/[Cl^-]_a$ and $[HCO_3^-]_i/[HCO_3^-]_a$ ratio at a respective HCO_3^- permeability) and a static ionic model (without dynamic changes in $[Cl^-]$ and $[HCO_3^-]$), the GHK equation will led to exactly identical results, if adequate values for the Cl^- and HCO_3^- parameters were used. For the present study we did not considered to analyze in addition how dynamic changes in $[Cl^-]_i$ or $[HCO_3^-]_i$ and different estimates of the Cl^-/HCO_3^- permeability ratio influence the effect of GABAergic currents. Therefore we decided to keep the simple Ohmic model of GABAergic currents for the revised version of the present study. However, in order to follow the reviewer’s suggestion, we discussed in the revised version of the manuscript to which $[Cl^-]_i$ the observed E_{GABA} values relate (line 506-512 and line 858-860).

I wish the author good luck and acknowledge that a lot of work went into the writing of this manuscript.

Thank you very much again.

Reviewer #3:

In neonatal brain, the GABAergic synaptic inputs exert excitatory effect unlike the matured adult brain where GABA causes inhibition. This reversed role of GABA is due to the higher intracellular Cl- concentration caused by the low expression levels of K+-Cl- cotransporters that extrude Cl- from the cell and the accumulation of Cl- due to NKCC1. This leads to the reversal potential for GABA (EGABA) to be less negative than the resting membrane potential. This study explores additional conditions required for GABAergic synaptic inputs to be excitatory. The key conclusions from the study are that for GABAergic inputs close to soma to be excitatory the threshold EGABA above which GABA becomes excitatory is close to the threshold for action potential. This threshold EGABA shifted to positive values. This threshold EGABA also depends on the spatial and temporal relation between GABA and AMPA inputs where it shifted to more negative values for AMPA inputs appearing after GABA input. The threshold EGABA shifted to values negative to threshold for action potential when AMPA synapses located proximally to the GABA input, while for distally located AMPA synapses the dendritic distance had only a minor effect on the threshold EGABA. Thus, the study shows that the excitatory effect of GABA is more complex than just the change in Cl- dynamics in neonatal brain.

Overall, I find this study very interesting and worth considering for publication in PLoS Comp Biology. However, a few issues remain that need to be addressed.

Thank you very much for your careful and positive evaluation.

In the first two postnatal weeks, the reversal potential for GABA is significantly more negative than the range predicted by the model. That is, the observed EGABA < -50 mV (see for example, Owens et al. J Neurosci. 1996, 6414-6423), whereas the model predicts that the threshold EGABA above which the GABA is excitatory is greater than or equal to -43.1 mV. By this account, the experimentall observed EGABA should always be inhibitory, which is not the case. Thus a detailed discussion of the model-predicted EGABA threshold in light of the experimental observations is needed

In accordance with the example suggested by the reviewer, E_{GABA} is in the hippocampus clearly below the RMP for the first 2 postnatal weeks, indicating an inwardly directed driving force and thus depolarizing GABAergic responses. During early phases the estimated E_{GABA} in hippocampal neurons was in the range of - 50 mV, and thus indeed below the range suggested from our in-silico study for E_{GABA}^{Thr}. However, we adapted the model parameters for our simulation to the recorded values for the RMP and the E_{AP}^{Thr} in immature hippocampal neurons. We assume, that the discrepancy between estimated E_{GABA}^{Thr} and the published E_{GABA} values is probably caused by the immanent inaccuracies in the determination of E_m and E_{AP}^{Thr} with conventional patch-clamp recordings, as elaborated by Tyzio et al. (Tyzio et al., 2003). Under consideration of their values for E_m, E_{AP}^{Thr} and E_{GABA}

one would expect a clear depolarizing and putative excitatory action. However, the principles derived from our in-silico study can be easily transferred to this situation by a linear shift in the absolute values for E_{GABA}^{Thr} and E_{AP}^{ST}, which were derived from conventional whole-cell recordings in immature hippocampal slices performed by us (Lombardi et al., 2018) to the more negative values suggested by Valeeva et al. (Valeeva et al., 2010) and Tyzio et al (Tyzio et al., 2003). We discussed this issue in detail in the revised version of the manuscript (line 513-532).

A model closely reproducing the shape of AP (that is, the sharp rise phase of AP) was previously developed by Naundorf et al. (2006) Nature 440(7087):1060–1063. Why is a new (more complex) formalism needed to model the actual shape of AP?

In order to follow the reviewer's suggestion, as well as a major concern of the second reviewer, we came to the conclusion that we will no longer use the not jet validated "modified Markov model" for the present study. Following your helpful suggestion, we instead used the AP model provided by Naundorf et al (2011) for all simulations included in the present manuscript. Please note that this change in the AP mechanisms led only to marginal quantitative changes in the results and had no effect on the general conclusions of the manuscript. The use of the model was introduced in line 150 and the details were explained in the methods section (line 780-814). The used parameters were provided in the Suppl. Table 4, and all experimental results, including all figures, were updated accordingly.

AMPA synapses are not developed in the first few postnatal days (see for example, Lohmann and Kessels, J. Physiol. (2014) 592, 13-31). Which raises the question about the validity of the simulations involving simultaneous AMPA and GABA inputs. The authors need to discuss the postnatal ages at which the different simulations performed in this study are relevant.

The onset of functional AMPA synapses during brain development is contro-versially discussed. While early reports bolster the concept of "GABA first, NMDA next, AMPA last" expression pattern (e.g. Ben-Ari's 1997 "Menage a trois" review), a variety of studies demonstrated that AMPA receptors are functionally relevant already at early postnatal ages. This is particularly demonstrated by the fact that the AMPA receptor antagonists CNQX/DNQX effectively inhibit early network activity (e.g. Wells et al., 2000, Lamsa 2000 or even Ben-Ari et al., 1989, on page 321). Therefore, we consider that the interaction of GABA and AMPA receptor mediated inputs is physiologically relevant at least in all (mice) postnatal stages. To follow the reviewer's concern, we mentioned this point in the revised manuscript in line 248-253.

Following are some minor concerns.

L 38: "immature brain of after neurological insults" should be "immature brain or after neurological insults".

We corrected this typo.

L 39: "depolarizations con contribute" should be "depolarizations can contribute"

We corrected this typo.

L 40: "to determine which amount of a GABAergic" should be "to determine what amount of a GABAergic".

We corrected this sentence (line 48).

L 78-79: The statement "However, it is important to consider that depolarizing GABA responses do not per se lead to excitatory effects" is not clear. GABA receptors are known to release Cl- from the cytoplasm in the neonatal brain, leading to excitation. Besides, if GABA responses are "depolarizing" then why won't they lead to "excitatory effects".

The notion that a depolarizing response lead to excitation, because it shifts E_m towards the AP threshold (E_{AP}^{Thr}), holds true only if the conductance changes underlying the depolarizing currents were not considered. In case of $GABA_A$ receptors, the most simple assumption considers that depolarizing GABA can mediate inhibition as long as E_{GABA} is below the AP threshold. The rationale behind this is that under this condition the Cl^-fluxes, which are outward during resting states, reverses whenever an EPSP crosses E_{GABA}. And above this voltage the GABA-receptor mediated Cl^- fluxes will dampen the EPSP amplitude and thus act inhibitory. In order to follow the reviewer's concern, we included this explanation in the revised version of the manuscript (Line 90-96).

L 169: The sentence "the distinct EAPThr parameters are virtually independent on the duration of" should be "the distinct EAPThr parameters are virtually independent of the duration of".

We corrected this sentence (Line 165).

L 242: "illustrated that gGABAThr showed a considerable less steep dependency" should be "illustrated that gGABAThr showed a considerably less steep dependency".

We corrected this sentence (line 222).

References for the Responses:

Ben-Ari Y, Cherubini E, Corradetti R, Gaiarsa J-L. Giant synaptic potentials in immature rat CA3 hippocampal neurones. J Physiol. 1989;416: 303–325.

Ben-Ari Y, Khazipov R, Leinekugel X, Caillard O, Gaiarsa JL. GABA(A), NMDA and AMPA receptors: A developmentally regulated "menage a trois." Trends in Neurosciences. 1997; 20: 523–529.

Lamsa K, Palva JM, Ruusuvuori E, Kaila K, Taira T. Synaptic GABA(A) activation inhibits AMPA-kainate receptor-mediated bursting in the newborn (P0-P2) rat hippocampus. J Neurophysiol. 2000;83: 359-366.

Lombardi A, Jedlicka P, Luhmann HJ, Kilb W. Giant depolarizing potentials trigger transient changes in the intracellular Cl - concentration in CA3 pyramidal neurons of the immature mouse hippocampus. Front Cell Neurosci. 2018;12:420.

Naundorf B, Wolf F, Volgushev M. Unique features of action potential initiation in cortical neurons. Nature. 2006;440: 1060–1063.

Tyzio R, Ivanov A, Bernard C, Holmes GL, Ben-Ari Y, Khazipov R. Membrane Potential of CA3 Hippocampal Pyramidal Cells during Postnatal Development. J Neurophysiol. 2003;90: 2964–2972.

Valeeva G, Abdullin A, Tyzio R, Skorinkin A, Nikolski E, Ben-Ari Y, et al. Temporal coding at the immature depolarizing gabaergic synapse. Front Cell Neurosci. 2010;4: 1–12.

Wells JE, Porter JT, Agmon A. GABAergic inhibition suppresses paroxysmal network activity in the neonatal rodent hippocampus and neocortex. J Neurosci. 2000;20: 8822–8830.

Wong B. Points of view: Color blindness. Nature Methods 2011;8:441.
